# Emergence of opinion leaders in reference networks

**DOI:** 10.1371/journal.pone.0193983

**Published:** 2018-03-26

**Authors:** Mariko I. Ito, Hisashi Ohtsuki, Akira Sasaki

**Affiliations:** 1 Department of Evolutionary Studies of Biosystems, SOKENDAI (The Graduate University for Advanced Studies), Hayama, Kanagawa, Japan; 2 Evolution and Ecology Program, International Institute for Applied Systems Analysis, Laxenburg, Austria; Peking University, CHINA

## Abstract

Individuals often refer to opinions of others when they make decisions in the real world. Our question is how the people’s reference structure self-organizes when people try to provide correct answers by referring to more accurate agents. We constructed an adaptive network model, in which each node represents an agent and each directed link represents a reference. In every iteration round within our model, each agent makes a decision sequentially by following the majority of the reference partners’ opinions and rewires a reference link to a partner if the partner’s performance falls below a given threshold. The value of this threshold is common for all agents and represents the performance assessment severity of the population. We found that the reference network self-organizes into a heterogeneous one with a nearly exponential in-degree (the number of followers) distribution, where reference links concentrate around agents with high intrinsic ability. In this heterogeneous network, the decision-making accuracy of agents improved on average. However, the proportion of agents who provided correct answers showed strong temporal fluctuation compared to that observed in the case in which each agent refers to randomly selected agents. We also found a counterintuitive phenomenon in which reference links concentrate more around high-ability agents and the population became smarter on average when the rewiring threshold was set lower than when it was set higher.

## 1 Introduction

In our society, individuals exchange information with one another [1–3]. Therefore, in many circumstances, interdependence of individuals’ opinions is inevitable [4, 5]. This idea is often considered in the literature of opinion formation, and the question about whether this interdependence leads to a preferable result in the population has been studied extensively [4–7].

One example of opinion interdependence is found in the reference behavior called the lead–follow relationship between financial analysts [8–13]. Financial analysts synthesize much of information and provide reports such as forecasts of a firm’s forthcoming earnings for investors. They revise their personal earnings forecasts at their own chosen time during the fiscal period. It has been suggested in the literature that less informative or less experienced analysts, to make their decisions more accurately, follow information developed by analysts called lead analysts, who announced their forecasts earlier [8, 10, 13–15].

Such lead–follow behavior is not necessarily limited to financial analysts —we expect to see it more generally in our society when each of us can refer to earlier opinions to make our decisions. In this paper, we consider the process of decision-making in a population in which individuals are mutually connected by reference links. Individuals are assumed to base their opinions on the majority of earlier opinions [16, 17] made by the referred individuals, i.e., we assume a directed network in which each node represents an individual making his/her decision, and each directed link refers to a reference relationship. Thus, the accuracy of an agent’s opinion depends on who the agent refers to. It is known that the majority-rule voting based on various (independent) opinions can result in higher accuracy than the one decided by an agent. This is known as collective intelligence [5, 17] (Table 1). However, it has also been shown that the correlation between opinions collected for a majority vote can decrease the advantage of collective intelligence [5, 6].

**Table 1:** An example showing the effect of collective intelligence.

The way the person with the highest accuracy (*p*_1_ = 0.75) makes a decision	Probability of answering a binary question correctly
Independent decision	0.75
Majority vote by referring to two other persons with lower accuracy (*p*_2_ = 0.7, *p*_3_ = 0.65)	0.785

Consider a society with three persons differing in their accuracy, or the probability, of giving the correct answer for a binary question when they make a decision alone. Suppose their accuracy is given by *p*_1_ = 0.75, *p*_2_ = 0.7, and *p*_3_ = 0.65. When person #1 (with accuracy *p*_1_ = 0.75) makes his/her decision independently, he/she gives the correct answer with probability *p*_1_ = 0.75. In contrast, if he/she refers to two other persons that have lower accuracy than him/herself for making a decision by the majority vote of all three, the probability of giving the correct answer is raised to 0.785 (calculated as *p*_1_*p*_2_*p*_3_ + (1 − *p*_1_)*p*_2_*p*_3_ + *p*_1_(1 − *p*_2_)*p*_3_ + *p*_1_ *p*_2_(1 − *p*_3_) = 0.785).

It should be natural to assume that each individual assesses the credibility of the referents and decides to either keep or stop following them accordingly. Thus, a reference link is rewired according to the accuracy of the referred agent, whose accuracy depends on who he/she refers to. Therefore, we need to consider the interaction between the change in opinion caused by network topology and the change of network topology induced by the opinion accuracy of the nodes. This idea is related to *adaptive* or *coevolutionary* networks, in which feedback loops between node dynamics and network topology are considered [18, 19]. There are a number of adaptive network models under various link-rewiring rules including ones that assume game interactions between nodes, such as the prisoner’s dilemma game and the minority game, in which a link represents a game interaction or reference. These links are discarded and rewired when the linked game partners are not preferable, when the linked advisers are not reliable, and so on [16, 18–24]. Some of these models show the emergence of heterogeneous structures in the evolved network, such as the scale-free degree distribution, in which a small number of individuals come to acquire a large number of degrees after repeated events of rewiring, even when starting from a homogeneous initial state [18, 20–24]. Some of these models also highlight macroscopic quantities, such as the ratio of cooperators in the population and the quality of propagated information (and “performance” in our model), that change through adaptive rewiring [18, 20, 21, 24] and show, in some cases, that the evolved network with a heterogeneous structure has better performance than the initial homogeneous network.

In our model, we focus on the accuracy of decision-making of each individual, which we call “performance”, in the evolved network. It is not clear whether the evolved network shows good performance. If the evolved network has high heterogeneity, so that some individuals, called leaders, receive a far larger number of reference edges than the others, then the opinions of agents in the network should be highly correlated. This enhanced correlation between opinions may harm the population performance in the long run. The opposite may be the case because the network structure, which is biased toward referring to more accurate agents, may improve the population performance. The performance in the evolved network should depend on the network’s structure.

In the reference relationship, we ask (1) what property is generated in the in-degree (the number of followers) distribution, that is, the correlation between the in-degree of an agent and its ability to solve a problem, in the evolved network and (2) whether the reference relationship in the evolved network leads to higher performance than the initial random network. To answer the questions listed above, we conducted extensive computer simulations and developed an analytical theory to explain the results obtained in the simulations.

In Section 2, we explain our model and how it incorporates the interaction between collective intelligence and network evolution. In Section 3, we show the results —there, we see the emergence of the heterogeneous structures with a nearly exponential in-degree distribution. We also evaluate the performance of the evolved network. We explain these results by analytical calculations. The discussion is in Section 4.

## 2 Materials and methods

### 2.1 Model

**Fig 1:**
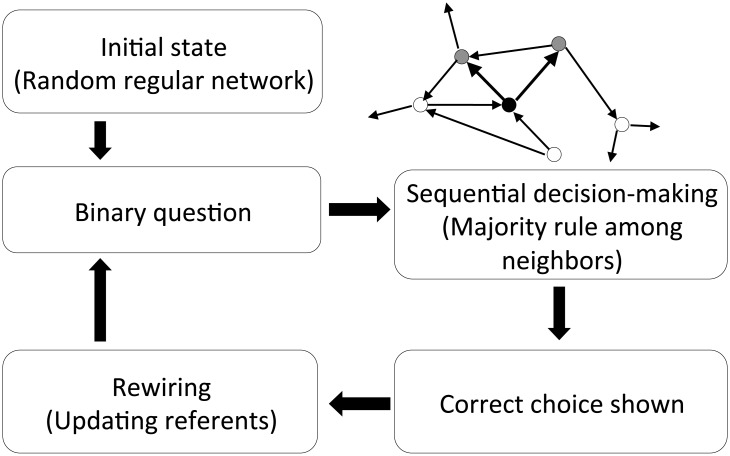
Procedure to update the network. The initial network is a random regular network. Starting from this initial network, we iterate sequential decision-making and rewiring of the reference links. Illustrated in the top-right part is a sample network, in which the closed dot represents the focal agent, the gray dots represent the agents referred to by the focal agents, and the arrows are reference links.

We consider a directed network made up of *N* nodes, each of which represents an agent who makes a decision. In this network, a directed link from node *i* to node *j* means that agent *i* refers to agent *j* when he/she makes a decision. If there is a directed link from *i* to *j*, we call agent *i* a *follower* of agent *j*, and agent *j* a *referent* of agent *i*. For each agent, the number of reference links from him/her is fixed to *M*. Let *a*_*ij*_ be the number of reference links from agent *i* to agent *j*—(*a*_*ij*_) is the adjacency matrix of the network (Table 2). By definition, $\sum_{j=1}^{N}a_{ij}=M$ and $\sum_{i=1}^{N}\sum_{j=1}^{N}a_{ij}=NM$. Here we allow for both self-loops and the overlap of links, i.e., *a*_*ii*_ is not necessarily 0 and *a*_*ij*_ can be more than 1. Each agent in our model repeatedly makes his/her decision while updating his/her referents by the rule explained later (Fig 1).

**Table 2:** Definition of symbols.

Symbol	Descriptions
*N*	Number of agents
*M*	Number of reference links from each agent
*a*_*ij*_	Number of reference links from agent *i* to agent *j*
*p*_*i*_	Ability of agent *i*
Π_*i*_	Probability that agent *i* gives a correct answer
$y_{t}^{ij}$	Evaluated performance of agent *j* by agent *i* at time *t*
*y*_0_	Initial value of $y_{t}^{ij}$ when agent *i* newly rewires to agent *j*
$I_{t}^{i}$	The random variable whose value is 1 (0) if agent *i* succeeded (failed) in giving a correct answer
*θ*	Rewiring threshold
*α*	The extent to which people attach importance to the current result against the history so far in the performance evaluation
$\bar{T_{i}}$	The mean duration that the agent *i* keeps his/her follower
$\bar{k}\left(p\right)$	The mean in-degree of an agent with ability *p*

In the initial condition, each agent refers to randomly selected *M* referents. In other words, a directed random regular graph with *M* out-degrees is used as the initial state of our model.

In our model, the same binary choice question is given to all agents. One of the choices is correct, and the other is wrong. We assume that agents vary in their probability of solving a problem correctly by themselves (i.e., without referring to others’ opinions). We call the probability *ability*. The ability of agent *i* is denoted by *p*_*i*_. For example, a question is given to financial analysts, such as whether the earning of a company in the next quarter increases, and all will know the correct answer at the end of the next quarter. After many trials, the analyst’s (say agent *i*’s) ability is calculated as the probability that he/she forecasted the correct outcome, that is represented by *p*_*i*_. For each question, all agents state their answers sequentially in a randomly determined order. Hereafter, we call specifically an actual stated choice an *answer*. Now, we explain how agent *i* states his/her answer. When agent *i*’s turn comes, he/she first sets his/her own choice for the given question without referring to those by his/her referents. The probability that this choice is correct is given by the agent’s ability, *p*_*i*_. In the next step, agent *i* puts his/her own choice together with the answers of referents that have already been stated and makes a final choice among those choice/answers according to the simple majority-rule. Agent *i* then states a final choice as his/her answer. For example, if agent *i* refers to agents *i*_1_, *i*_2_, *i*_3_, *i*_4_ and *i*_5_, and agents *i*_1_ and *i*_2_ have already stated their answers, agent *i* collects the answer from *i*_1_ and *i*_2_’s along with his/her choice and states the majority among these three. In the case of a tie in applying the majority-rule, agent *i* tosses a coin to decide which choice he/she states. In the case where *a*_*ii*_ ≥ 1, we regard that agent *i*’s choice has a weight *a*_*ii*_ + 1 in *i*’s majority-rule voting. Similarly, when *a*_*ij*_ ≥ 2(*i* ≠ *j*), we regard that agent *j*’s answer has a weight *a*_*ij*_ in *i*’s majority-rule voting. Since agent *i* incorporates other agents’ answers, it is clear that agent *i*’s ability *p*_*i*_ is different from the probability with which agent *i* actually states a correct answer, Π_*i*_. We denote Π_*i*_ as the *performance* of agent *i*.

After all the agents state their answers, the correct answer to the question is given. We assume that each agent monitors and evaluates the performance of his/her referents, as explained later. Each agent breaks the link to the referent if his/her evaluated performance falls below a certain threshold and rewires it to a randomly selected agent excluding referents that are kicked off in this step. The assumption that the newly selected agents are determined randomly is based on the idea that we cannot know the performance of strangers a priori.

We iterate decision-making and rewiring as explained above. In each iteration step *t*, the *evaluated performance* of referent *j* by agent *i*, denoted as $y_{t}^{ij}$, is updated as follows. When agent *i* newly acquires referent *j* at time *t*_*s*_, his/her initial level of estimated performance, $y_{t_{s}}^{ij}$, is set to *y*_0_ for any pair of *i* and *j*. Let $I_{t}^{j}$ be a variable whose value of 0 represents the failure of agent *j* to give a correct answer, and a value of 1 represents the agent’s success at the iteration time *t*. We assume that the evaluated performance of agent *j* by agent *i* at iteration time *t*, $y_{t}^{ij}$, is updated recursively by

$$\begin{array}{c} y_{t}^{ij}=\left(1-\alpha \right)y_{t-1}^{ij}+\alpha I_{t}^{j}. \end{array}$$ (1)

Here, *α* describes the extent to which people in the society attach importance to the current result as compared to the referent’s past. We also assume that all agents adopt the same rewiring threshold to kick-off referents, *θ*. The threshold represents severity of assessment in the society. For example, if the threshold is low, people in the society are generous when they evaluate their referents.

### 2.2 Simulation conditions

We conducted agent-based simulations using parameters *N* = 100, *M* = 5, and *α* = 0.1. We assume that the ability of agents, *p*_*i*_’s, are uniformly distributed in the range of 0.5 to 0.75 by setting *p*_*i*_ = 0.5 + 0.25*i*/*N* for *i* = 0, 1, …, *N* − 1. Note that the ability of each agent does not change throughout a simulation run. The initial evaluated performance *y*_0_ is set to 0.625, which is nearly equivalent to the mean ability in the population. We limit the range of the rewiring threshold *θ* in 0.5 ≤ *θ* < *y*_0_ = 0.625. The lower limit for *θ*, 0.5, is only the accuracy of a coin-toss. The upper limit *y*_0_ is set for the following reason—if we set the threshold greater than this upper limit, the initial performance of a new referent is always evaluated lower than the threshold.

**Fig 2:**
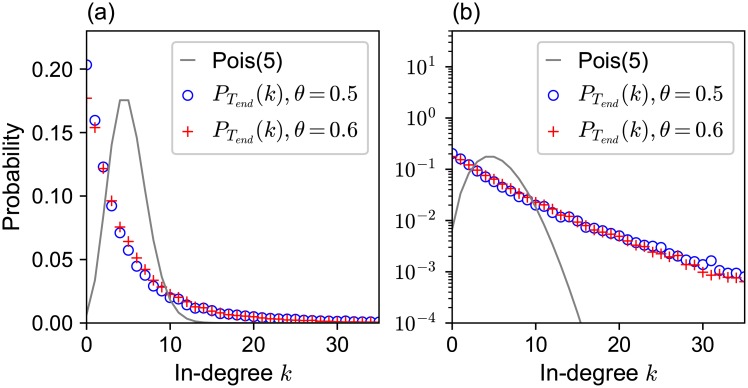
The distribution of the number of followers (in-degree) in the initial and evolved networks. (a) The solid line is the initial Poisson distribution with a mean of 5. The markers (the circle and +) denote the in-degree distributions of the evolved network with rewiring thresholds *θ* = 0.5 and 0.6, respectively, obtained over 500 independent runs of our simulation. The in-degree distributions in the evolved networks are significantly different from the initial condition, showing much higher heterogeneity in in-degrees. (b) The same as (a) except that the vertical axis is logarithmically scaled. We can observe the approximately exponential tails in the evolved networks.

At the initial state, the network is a directed random regular network with out-degree *M* = 5, so the in-degree distribution of each agent is expected to obey the binomial distribution with parameters *NM* (the total number of reference links in the population) and 1/*N* (the probability that a particular individual is chosen as a referent) and is approximated by the Poisson distribution with mean *M*, because *N* ≫ 1 (Fig 2).

In our simulation, a set of sequential decision-makings of agents, followed by the rewiring of reference links, constituted the events in a unit of time, which repeats itself until the “evolved network” at *t* = *T*_end_ = 20,000 was reached. For each parameter set, the simulations were repeated 500 times. We calculated the frequency of agents having in-degree *k* at each time step *t* and averaged them over 500 independent runs. *P*_*t*_(*k*) denotes the averaged frequency of agents having in-degree *k* at time *t*, which must depend on the rewiring threshold *θ*. We regard $P_{T_{\text{end}}}\left(k\right)$ as the in-degree distribution in the evolved network. We evaluated the performance of each agent for each simulation run in the evolved network by averaging the number of correct answers stated in the last *T* = 100 time steps (i.e., the performance of agent *i* is the average of $I_{t}^{i}$ over *T*_end_ − *T* + 1 ≤ *t* ≤ *T*_end_), at which point we assumed that the network has reached an equilibrium state. The average over 500 independent runs was then calculated and regarded as the mean performance of agent *i*, Π_*i*_. Therefore, the definition of Π_*i*_ is $\Pi_{i}=\bar{\sum_{t=T_{\text{end}}-T+1}^{T_{\text{end}}}I_{t}^{i}/T}$, where the overline represents the average over 500 independent runs. We also calculated the mean group performance and its standard deviation. For each single run, we regarded $\sum_{i=1}^{N}I_{t}^{i}/N$ as the group performance at time *t* and calculated the mean and the standard deviation (SD) of ${\left\{\overset{N}{\underset{i=1}{\sum }}I_{t}^{i}/N\right\}}_{T_{\text{end}}-T+1\leq t\leq T_{\text{end}}}$, that is, for the last *T* = 100 time steps. Then we took their average over 500 independent runs to evaluate the group performance and its fluctuation.

## 3 Results

### 3.1 The heterogeneity in the in-degree distribution

The in-degree distribution $P_{T_{\text{end}}}\left(k\right)$ in the evolved network significantly differed from the initial Poisson distribution for the random network (Fig 2). As the in-degrees in the evolved network were distributed approximately exponentially, there were a few nodes that had much larger in-degrees than the mean. In other words, high heterogeneity in the number of followers evolved through the adaptive rewiring process. The agents attracting many followers can be interpreted as “opinion leaders” in our model.

**Fig 3:**
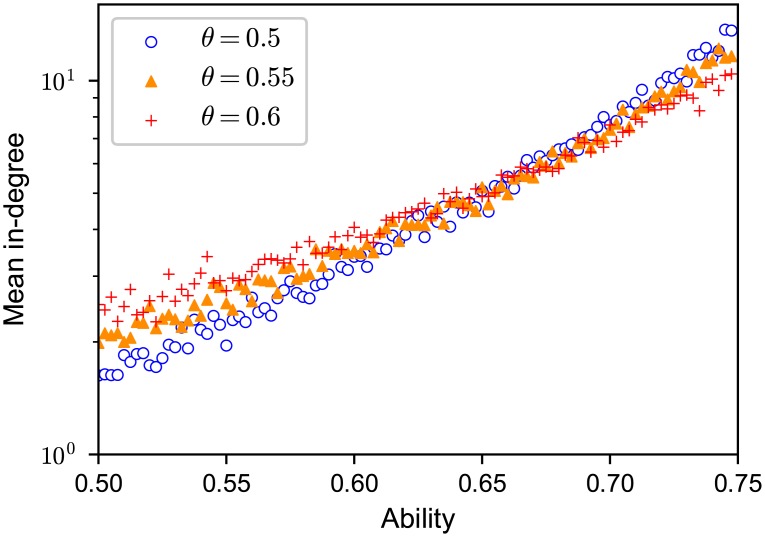
Semi-log plot of the mean in-degree of an agent versus his/her ability in the evolved network obtained by simulations. Different symbols represent the results for varying *θ*. The mean in-degree increases approximately exponentially with ability, and slopes are steeper when we set the threshold lower. The vertical axis is scaled logarithmically.

The mean in-degree $\bar{k}\left(p_{i}\right)=\bar{\sum_{j=1}^{N}a_{ij}}$ of agent *i* with ability *p*_*i*_, that is the mean number of followers of agent *i*, increased exponentially with *p*_*i*_ for each rewiring threshold *θ* (Fig 3).

**Fig 4:**
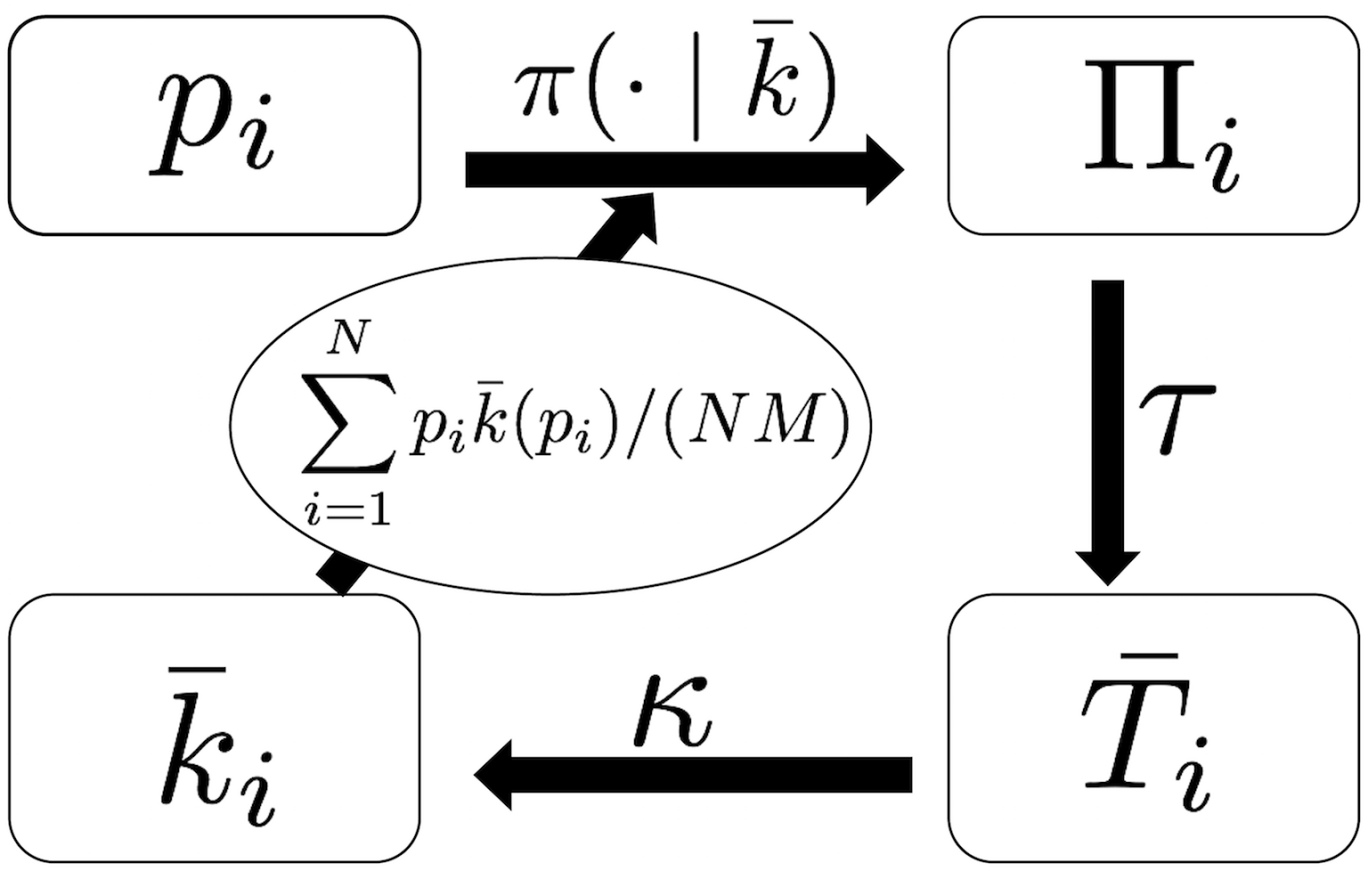
The schematic diagram for the relationship between the ability of agent *i*, *p*_*i*_, his/her mean performance Π_*i*_, the mean duration that he/she is kept linked by a follower ${\bar{T}}_{i}$, and his/her mean in-degree ${\bar{k}}_{i}$ in the equilibrium state. The mean performance can be derived approximately by the ability *p*_*i*_ and the mean in-degree function $\bar{k}\left(\cdot \right)$ through the function $\pi (\cdot |\bar{k})$. The mean duration that the agent is kept linked by a follower is obtained by the mean performance (the function *τ*). Then, the mean in-degree is obtained by the mean duration the agent is kept linked by a follower (the function *κ*).

We were able to obtain an approximation equation which $\bar{k}\left(p_{i}\right)$ satisfies in the equilibrium state. Its derivation, which we will describe in detail in Sections 3.2 to 3.5, is illustrated as follows (Fig 4). Suppose we know $\bar{k}\left(p_{i}\right)$. In the equilibrium state, the probability that a randomly sampled reference link from the population is referring to agent *i* should be proportional to the mean number of followers of agent *i* and is expressed as

$$\begin{array}{c} \text{Pr}.[\text{agent}\;i\;\text{is}\;\text{being}\;\text{referred}]=\frac{\bar{k}\left(p_{i}\right)}{\sum_{j=1}^{N}\bar{k}\left(p_{j}\right)}=\frac{\bar{k}\left(p_{i}\right)}{NM}. \end{array}$$ (2)

Based on these probabilities, we can derive the approximation of the mean performance Π_*i*_ of agent *i*, who has ability *p*_*i*_ (see Section 3.2). Given Π_*i*_, the mean duration $\bar{T_{i}}$ that the agent is kept linked by a follower is calculated in Section 3.3. Finally, given $\bar{T_{i}}$, the mean number of followers $\bar{k}\left(p_{i}\right)$ of agent *i* is derived in Section 3.4. Therefore, we obtained an implicit equation of $\bar{k}\left(p_{i}\right)$ in the evolved network. This equation of $\bar{k}\left(p_{i}\right)$ explains why agents with higher ability have acceleratingly more followers. Since we cannot solve this equation for $\bar{k}\left(p_{i}\right)$, we performed an iterative approximation method to numerically obtain $\bar{k}\left(p_{i}\right)$ against *p*_*i*_. This procedure is explained in Section 3.5. The results obtained by this numerical calculation agree well with the simulation results.

### 3.2 The relationship between the mean performance of an agent and his/her ability

**Fig 5:**
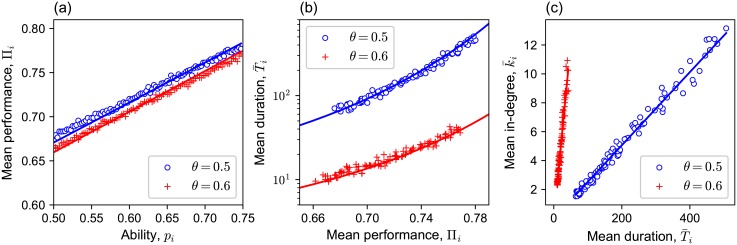
The mean performance Π_*i*_ of an agent versus his/her ability *p*, the mean duration ${\bar{T}}_{i}$ that the agent is kept by a follower versus the agent’s mean performance Π_*i*_, and the mean in-degree ${\bar{k}}_{i}$ of an agent versus the mean duration ${\bar{T}}_{i}$ that the agent is kept by a follower in the evolved network. (a) The mean performance Π_*i*_ of an agent versus his/her ability *p* in the evolved network for each threshold *θ* (the circle and + for thresholds 0.5 and 0.6). The solid lines show the analytical results. The mean performance increases linearly with ability. (b) The semi-log plot of the mean duration ${\bar{T}}_{i}$ that the agent is kept by a follower versus the agent’s mean performance Π_*i*_ for each threshold in the evolved network (the circle and + for thresholds 0.5 and 0.6). The solid lines show the analytical results. The mean duration increases nearly exponentially with the mean performance. (c) The mean in-degree ${\bar{k}}_{i}$ of an agent versus the mean duration ${\bar{T}}_{i}$ that the agent is kept by a follower for each threshold in the evolved network (the circle and + for thresholds 0.5 and 0.6). The solid lines show the analytical results. The mean in-degree is proportional to the mean duration.

We calculated the mean performance of agent *i* in the evolved network, $\Pi_{i}=\bar{\sum_{t=T_{\text{end}}-T+1}^{T_{\text{end}}}I_{t}^{i}/T}$, as explained in Section 2. For each threshold, the mean performance of an agent increased linearly with his/her own ability (Fig 5(a)). This is because the probability that agent *i* with ability *p*_*i*_ gives a correct answer is given by the sum of two terms,

$$\begin{array}{cc} & \Pi_{i}=\left(1-p_{i}\right)\text{Pr}.\left[\left(\text{the}\;\text{number}\;\text{of}\;\text{referents}\;\text{who}\;\text{gave}\;\text{correct}\;\text{answers}\right)\geq s/2+1\right]\\ & \;+p_{i}\text{Pr}.\left[\left(\text{the}\;\text{number}\;\text{of}\;\text{referents}\;\text{who}\;\text{gave}\;\text{correct}\;\text{answers}\right)\geq s/2\right], \end{array}$$ (3)

when the number of referents of agent *i* who have stated their answers before agent *i* stated his/her own, which we denote by *s*, is even. Note that here, we neglect self-loops or overlaps in the reference links to simplify our approximation. Also note that the expression of Π_*i*_ becomes a slightly complicated when *s* is odd (Section A in S1 Text) since there are *s* + 1 answers/choice including his/her own and we have to consider the tie of the number of answers/choice when the majority-rule is applied. However, Π_*i*_ can again be described as a linear function of the ability *p*_*i*_ in both cases where *s* is odd and where there are self-loops or overlaps in the links (Section A in S1 Text).

Note that each term Pr.[⋅] in the equations above depends on the ability and the performance of agents who the focal agent refers to; therefore, Pr.[⋅] depends on the distribution of the *ability of referents*, which means the ability of those who are referred to by others, not on the ability of random agents.

We can derive an approximate formula for the slope and the intercept of the linear dependence of Π_*i*_ on *p*_*i*_ (Section A in S1 Text), which agrees well with the simulation results (Fig 5(a)). For later use, let us formally denote this relation as $\Pi_{i}=\pi \left(p_{i}|\bar{k}\right)$, where $\pi (\cdot |\bar{k})$ maps *p*_*i*_ to Π_*i*_, which itself depends on $\bar{k}$. The approximated slope and intercept depend on the mean ability of referents $\sum_{i=1}^{N}p_{i}\bar{k}\left(p_{i}\right)/\left(NM\right)$, which is a value that represents the distribution of referents’ ability.

### 3.3 Relationship between the mean duration for which a referent is kept linked by a follower and his/her performance

In our model, each agent monitors the performance of his/her referents and stops referring to them when the evaluated performance falls below a rewiring threshold. Thus, the higher his/her referent’s performance is, the longer duration that he/she keeps his/her follower. We herein examine how the duration that an agent is kept referred by a follower is related to the agent’s performance.

The mean duration that an agent is kept referred by a follower in the evolved networks increased approximately exponentially with his/her performance (Fig 5(b)): $\bar{T_{i}}\propto \text{exp}\left(\beta \Pi_{i}\right)$, where $\bar{T_{i}}$ is the expected duration that agent *i* keeps his/her follower, Π_*i*_ is the performance of agent *i*, and *β* is a positive constant.

This relationship between a referent’s performance and the mean duration for which the referent is kept linked by a follower is derived analytically. As explained in the Method section, an agent’s evaluation *Y*_*t*_ of the performance of his/her referent is updated depending on whether the referent’s *t*-th answer was correct (*I*_*t*_ = 1) or not (*I*_*t*_ = 0), as follows:

$$\begin{array}{c} Y_{t}=\left(1-\alpha \right)Y_{t-1}+\alpha I_{t},\;t=1,2,\ldots , \end{array}$$ (4)

where the initial evaluation was set to *Y*_0_ ≡ *y*_0_. If the actual performance of the referent is Π, which its follower does not know, *I*_*t*_ (*t* = 1, 2, …) are mutually independent random variables each of which takes a value of 1 with a probability of Π, and a value of 0 with a probability of 1 − Π. The sequence {*Y*_*t*_ ∣ *Y*_0_ = *y*_0_} then forms a stochastic process. Given *Y*_0_ = *y*, we defined the expected time duration to the time when the evaluated performance hit *θ* for the first time, *T*_Π_(*y*), as *T*_Π_(*y*) ≡ E[min{*t* ∣ *Y*_*t*_ ≤ *θ*} ∣ *Y*_0_ = *y*]. *T*_Π_(*y*) is the expected first hitting time to the threshold *θ* of the stochastic process {*Y*_*t*_ ∣ *Y*_0_ = *y*}. Then *T*_Π_(*y*) satisfies the recurrence equation:

$$\begin{array}{ccc} T_{\Pi }\left(y\right) & = & 1+\Pi T_{\Pi }(\alpha +\left(1-\alpha \right)y)\\ & & +\left(1-\Pi \right)T_{\Pi }(\left(1-\alpha \right)y),\;y>\theta , \end{array}$$ (5)

and

$$\begin{array}{c} T_{\Pi }\left(y\right)\equiv 0,\;y\leq \theta . \end{array}$$ (6)

Eq (5) is derived as follows: if *y* > *θ*, the referent is kept linked to the next time step; hence, the addition of 1 in the first term on the right-hand side of (5). In the case where the referent gave the correct answer with probability Π, the evaluated performance changes from *y* to (1 − *α*)*y* + *α*, and the expected duration after the transition is *T*_Π_((1 − *α*)*y* + *α*). The last term is similarly derived for the case of failure. Eq (6) simply states that *T*_Π_(*y*) equals 0 if the evaluation *y* is already less than or equal to the threshold.

The mean duration that agent *i* with performance Π_*i*_ is linked from a follower, $\bar{T_{i}}$, is then defined by *T*_Π_(*y*) as follows:

$$\begin{array}{c} \bar{T_{i}}=T_{\Pi_{i}}(y_{0})\left(\equiv \tau \left(\Pi_{i}\right)\right), \end{array}$$ (7)

The symbol *τ* in Eq (7) denotes the function that maps Π_*i*_ to $\bar{T_{i}}$ (Fig 4). Note that $\bar{T_{i}}$ is greater than 0 since *θ* < *y*_0_. We solved recurrence Eqs (5) and (6) numerically and obtained the mean time *T*_Π_(*y*) until which the evaluated performance of a referent with the initial evaluated performance *y* and the actual performance Π hits the threshold *θ* for the first time (see Section D in S1 Text for the numerical procedure to obtain the mean hitting time). This then led to the mean duration of reference $\bar{T_{i}}$ defined in Eq (7). The analytical formulas (5)–(7) agree well with the simulation results (Fig 5(b)).

### 3.4 Relationship between an agent’s mean number of followers and his/her ability

Here, we derive the mean number of followers or the in-degree $\bar{k}\left(p_{i}\right)$ of agent *i* as a function of his/her ability *p*_*i*_.

The probability that an agent is chosen as a new referent for each rewiring event is the same as those for all the others’, because each agent rewires its link to a randomly selected agent after he/she kicks off a referent. Thus, the expected number of reference links that agent *i* receives in the evolved network is proportional to the mean lifetime of a link to agent *i*, $\bar{T_{i}}$. Therefore, the mean in-degree $\bar{k}\left(p_{i}\right)$ of agent *i* can be expressed as a function of $\bar{T_{i}}$ as follows:

$$\begin{array}{c} \bar{k}\left(p_{i}\right)=\frac{\bar{T_{i}}}{\sum_{j=1}^{N}\bar{T_{j}}}NM\left(\equiv \kappa \left(\bar{T_{i}}\right)\right). \end{array}$$ (8)

The symbol *κ* in Eq (8) denotes the function that maps $\bar{T_{i}}$ to $\bar{k}\left(p_{i}\right)$. This expression of $\bar{k}\left(p_{i}\right)$ agrees well with the simulation data (Fig 5(c)). The reason why $\bar{k}$ depends (only) on the ability *p*_*i*_ of agent *i* is that *τ*(Π_*i*_) is a function of the agent’s performance, Π_*i*_, and $\pi (p_{i}|\bar{k})$ is a function of *p*_*i*_. See Section C in S1 Text for a more formal derivation of $\bar{k}\left(p_{i}\right)$ from a master equation for the probability distribution of the performance of a referred agent.

As noted in the last section and shown in Fig 5(a) and 5(b), the mean duration that the reference to agent *i* is kept linked by a follower increases roughly exponentially (but actually slightly faster than exponential) with his/her performance Π_*i*_, and Π_*i*_ increases linearly with his/her ability *p*_*i*_, resulting in an roughly exponential relationship between $\bar{T_{i}}$ and *p*_*i*_: ${\bar{T}}_{i}\propto e^{\beta^{\prime }p_{i}}$. Therefore, the mean in-degree of agent *i* also increases roughly exponentially with his/her ability:

$$\begin{array}{c} \bar{k}\left(p_{i}\right)=\kappa \circ \tau \circ \pi \left(\cdot |\bar{k}\right)\left(p_{i}\right)\approx \left(\text{const.}\right)\times e^{\beta^{\prime }p_{i}}, \end{array}$$ (9)

where ∘ in Eq (9) is the composition of functions.

**Fig 6:**
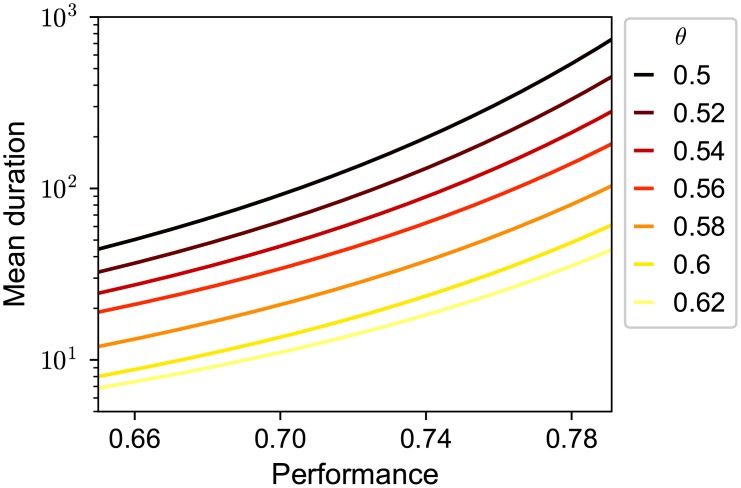
Semi-log plot of the mean duration to be kept by a follower versus performance for each threshold value. The mean duration is calculated analytically as shown in Section 3.3. The color of the lines ranges from dark to light as the threshold increases.

As we discussed above, the mean duration of a link targeted to an agent versus his/her performance is key to predicting how many followers an agent with a given ability can obtain. We show the duration versus performance for various thresholds, which we derived analytically, in Fig 6.

### 3.5 Numerical calculation to obtain $\bar{k}\left(p_{i}\right)$

Since Eq (9) is implicit in $\bar{k}\left(p_{i}\right)$, we solve it for $\bar{k}\left(p_{i}\right)$ by an iterative approximation method as follows. First, we set ${\bar{k}}^{\left(0\right)}\left(p_{i}\right)=M$ for all *p*_*i*_ as an initial condition (0-th step) of the iterative method. Its *n*-th iteration counterpart is ${\bar{k}}^{\left(n\right)}\left(p_{i}\right)$. Here is the procedure to obtain ${\bar{k}}^{\left(n+1\right)}\left(p_{i}\right)$ from ${\bar{k}}^{\left(n\right)}\left(p_{i}\right)$. By assuming that a randomly chosen reference link from the population is directed to agent *i* with a probability of ${\bar{k}}^{\left(n\right)}\left(p_{i}\right)/\left(NM\right)$, the mean performance $\Pi_{i}^{\left(n\right)}$ of agent *i* is obtained as explained in Section 3.2. Given the mean performance $\Pi_{i}^{\left(n\right)}$ of agent *i*, the mean duration ${\bar{T_{i}}}^{\left(n\right)}$ that the agent is kept linked by a follower is calculated as explained in Section 3.3. Then ${\bar{k}}^{\left(n+1\right)}\left(p_{i}\right)$ is calculated as

$$\begin{array}{c} {\bar{k}}^{\left(n+1\right)}\left(p_{i}\right)=\frac{{\bar{T_{i}}}^{\left(n\right)}}{\sum_{j=1}^{N}{\bar{T_{j}}}^{\left(n\right)}}NM, \end{array}$$ (10)

as explained in Eq (8) in Section 3.4. In other words, we derive ${\bar{k}}^{\left(n+1\right)}\left(p_{i}\right)$ from ${\bar{k}}^{\left(n\right)}\left(p_{i}\right)$, by

$$\begin{array}{c} {\bar{k}}^{\left(n+1\right)}\left(p_{i}\right)=\kappa \circ \tau \circ \pi \left(\cdot |{\bar{k}}^{\left(n\right)}\right)\left(p_{i}\right). \end{array}$$ (11)

**Fig 7:**
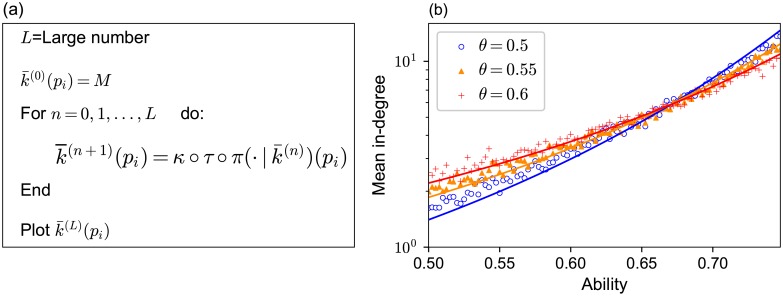
The derivation of the mean in-degree by the iterative approximation method. (a) The procedure of the iterative approximation method for obtaining the relationship between the mean in-degree and ability. (b) Curves obtained from (a) are shown against plots of simulation data.

We repeated this recurrence evaluation until when $\sum_{j=1}^{N}{\left({\bar{k}}^{\left(n+1\right)}\left(p_{i}\right)-{\bar{k}}^{\left(n\right)}\left(p_{i}\right)\right)}^{2}$ became smaller than 10^−5^ (Fig 7(a)). This predicted relationship (solid curves in Fig 7(b)) agrees well with the simulation results (markers in Fig 7(b)).

### 3.6 Group performance in the evolved network

**Fig 8:**
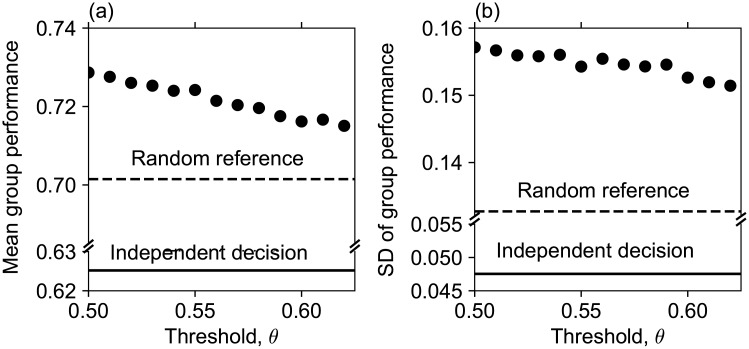
Mean and SD of group performance. (a) Mean group performance in the evolved network for each threshold. The dashed line represents the group performance in the initial random network, and the thick horizontal line representss the group performance in the case of independent decision-making. The mean group performance in the evolved network is higher than that in the random network for all thresholds, and it declines with increasing threshold. (b) The standard deviation SD of group performance versus threshold. The dashed line represents the SD in the random network, and the thick horizontal line represents the SD in the case of independent decision-making. The SD of group performance in the evolved network is also higher than that in the random network for all thresholds, and it gradually declines with increasing threshold.

Studying the group performance, $\sum_{i=1}^{N}I_{t}^{i}/N$, in the evolved networks is another objective of our paper. The temporal mean (Fig 8(a)) and the temporal standard deviation, SD (Fig 8(b)) of the group performance in the evolved networks were decreasing functions of the rewiring threshold. It is interesting that the stricter the agent’s evaluation threshold is for kicking off referents, the worse the long-term group performance is. For comparison, we showed in Fig 8(a) and 8(b) the mean and the SD when all agents choose referents randomly (random reference), as in the initial network state prior to adaptive rewiring (dashed lines in Fig 8(a) and 8(b)). We also added those measures in the case where all agents make their decision independently without constructing a network (independent decision; thick horizontal lines in Fig 8(a) and 8(b)). The difference between the thick line and the dashed line represents the effect of collective intelligence (decision-making through majority-rule). The difference between the dashed line (random reference network) and the dots (evolved network after adaptive rewiring) represents the effect of adaptive rewiring of the reference network on group performance, i.e., adaptive rewiring generates a centralized network with preferred connections towards high performance agents. The mean group performance was lowest when agents made decisions by themselves, which is improved by collective intelligence with randomly assigned referents and further improved by adaptive rewiring based on the performance evaluation. Among adaptively rewired networks, those with lower kick-off performance thresholds (i.e., with more generous kick-offs) had higher group performance. We see that the SD of group performance also increased in the same order as the mean group performance in this comparison, i.e., the group performance fluctuated more when the mean group performance became higher.

**Fig 9:**
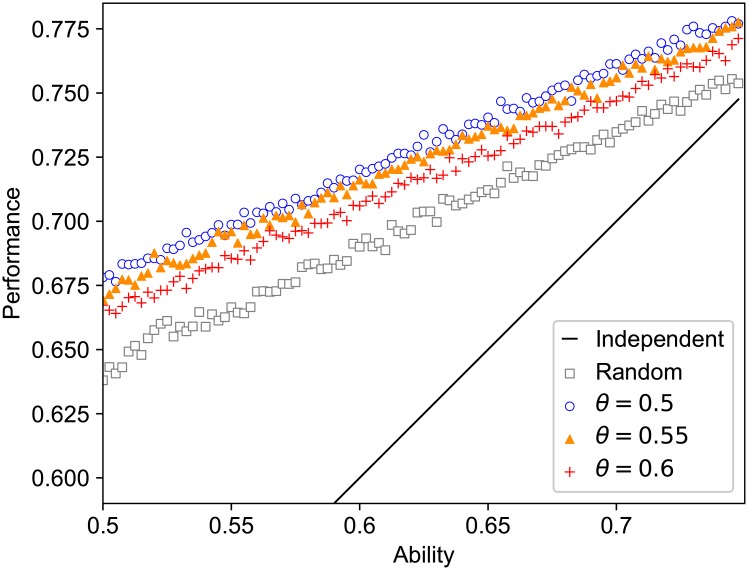
Mean performance of each agent versus his/her ability. A circle, triangle, and + mark the the mean performance versus ability for thresholds of *θ* = 0.5, 0.55 and 0.6 in the evolved network. A black square represents the random network. The solid diagonal line represents the case where performance is equal to ability. Even in the random network, all agents improve their accuracy (the mean performance is higher than the ability for each agent), and low-ability agents can particularly greatly improve it. The lower we set the threshold, the higher the mean performance becomes for each agent.

The mean performance of each agent, $\bar{\sum_{t=T_{\text{end}}-T+1}^{T_{\text{end}}}I_{t}^{i}/T}$, against his/her ability in the evolved networks was compared to both those in the cases of independent decision and of random reference (Fig 9). As in the group performance, for a fixed ability value of an agent, the mean performance was the lowest when the agent made decisions independently of others (solid line in Fig 9), which is improved by collective intelligence with randomly assigned referents (squares in Fig 9) and further improved by adaptive rewiring (circles, triangles and + in Fig 9). The effect of the rewiring threshold on the mean performance of each agent was similar to the effect of the threshold on the mean group performance: a looser kick-off threshold led to a higher performance. Fig 9 illustrates that the difference between independent decisions and majority voting, either adaptive or not, was reflected in both the slope and the intercept of the performance–ability relationship. However, the differences between the random and adaptive networks and those among different rewiring thresholds were reflected only in their intercepts. This leads to an interesting observation: agents with a lower ability were merited the most in their performance by collective intelligence, and the performance of all agents was improved fairly well by the adaptive rewiring irrespective of their ability.

To summarize, performance in the evolved network improved compared with the initial random network or the case of independent decision-making. However, the group performance fluctuated more in the evolved networks, and even more in those networks with higher mean group performance. This implies that a highly “intelligent” population with improved performance, though biased with reference to high-ability agents, can be at risk of a temporal crash in group performance.

### 3.7 The effect of threshold on the unevenness in in-degrees

**Fig 10:**
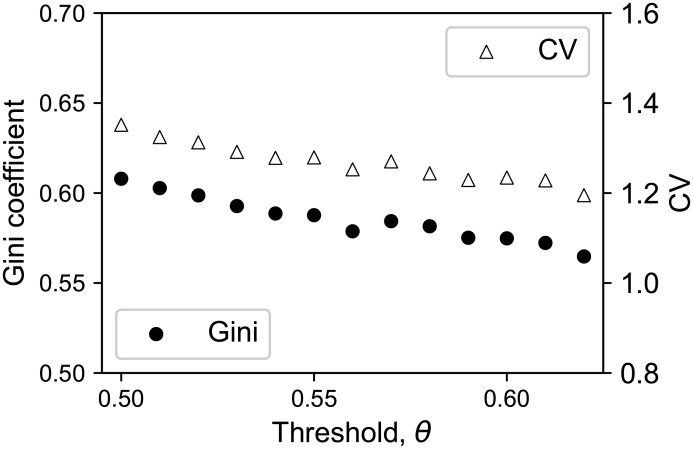
The Gini coefficient and the coefficient of variance (CV) of in-degree distribution. The Gini coefficient (circle) and the coefficient of variance (triangle) (CV) of in-degree distribution versus threshold. Both of these indices represent the strength of heterogeneity in in-degrees, where higher values mean stronger heterogeneity. Both the Gini coefficient and the CV decline with increasing threshold. Herein, the Gini coefficient *G* can be calculated as $G=\sum_{i,j=1}^{N}\left|k_{i}-k_{j}\right|/\left(2N^{2}\bar{k}\right)$, where *k*_*i*_ is the in-degree of agent *i* and $\bar{k}$ is the mean in-degree [25].

The threshold *θ* used for rewiring, which stands for the severity of assessment, affected the evolved network in the following aspects. First, thresholds affected the strength of heterogeneity in in-degrees among agents. We examined two heterogeneity measures of in-degree distribution at time *T*_*end*_, the Gini coefficient ($G=\sum_{i,j=1}^{N}|k_{i}-k_{j}|/(2N^{2}\bar{k}$), where *k*_*i*_ and *k*_*j*_ are the in-degrees of agent *i* and *j* respectively, and $\bar{k}$ is the mean in-degree of the population [25]), and the coefficient of variation of in-degrees (CV $=\sqrt{\text{Var}(k)}/\bar{k}$). They showed substantial dependence on the threshold *θ* (Fig 10). The Gini coefficient and the CV are indices that are originally used to represent the inequality in the distribution of wealth in a society. Here “the number of followers” (or “in-degree”) plays a role of “wealth”. We measured the inequality in the number of followers by using these indices. Higher values of these indices mean strong heterogeneity in in-degrees. In Fig 10, for both indices, the lower is the threshold for rewiring, the higher are the values of these indices. Therefore, both of these two indices show that a lower threshold for rewiring generates stronger inequality in the evolved in-degree distribution.

**Fig 11:**
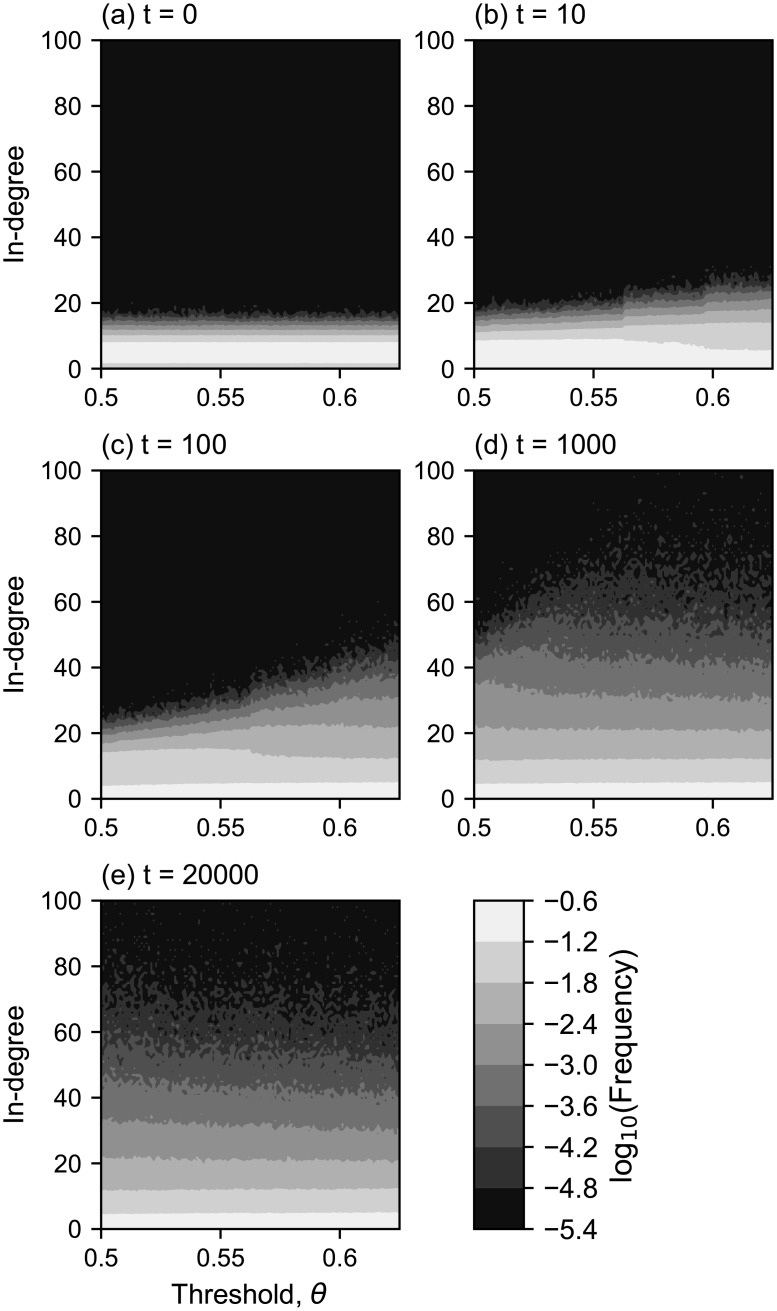
The in-degree distributions for each threshold at the *t* = 0, 10, 1,000 and 20,000(*T*_end_). The in-degree distributions for each threshold at the random network (*t* = 0) and at times 10, 100, 1,000 and 20,000 (*T*_end_) are shown in (a), (b), (c), (d), and (e), respectively. For each panel, the horizontal axis corresponds to the threshold, and the vertical axis represents the in-degree. The log_10_(*frequency*) is shown by the gray scale, so when we see a vertical section at a threshold *θ*, we can see an in-degree distribution for the threshold *θ*, such as the one shown in Fig 2(b).

The threshold *θ* also affected the time needed for the system to reach the equilibrium state (Fig 11). In Fig 11, the lighter colors represent a higher frequency of agents with a given in-degree *k* (ordinate) against a threshold *θ* (abscissa) at time *t*. We can see that the class of individuals with higher *k* grows faster for higher rewiring thresholds.

**Fig 12:**
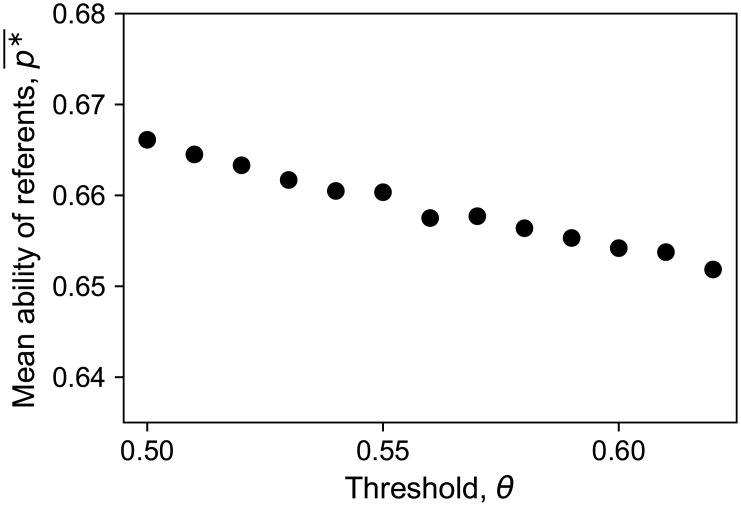
The mean ability of referents in the evolved network versus threshold. The lower we set the threshold, the more the mean ability of referents increases.

The exponential increase of the mean in-degree $\bar{k}\left(p\right)$ against ability *p* is also affected by the threshold *θ* (Fig 3). This nonlinearity in $\bar{k}\left(p\right)$ became stronger as the rewiring threshold *θ* decreased. Our analytical formula for the relationship between an agent’s mean in-degree and ability (Eqs (8) and (9)) shows that the strongly biased links towards the agents of high ability is due to the nonlinear dependence of the mean duration that an agent keeps a follower on their performance. We have already seen that the extent to which the mean duration increased with performance was stronger for lower thresholds (Figs 5(b) and 6). These results can be also seen in Fig 12, which shows that the mean ability of referents (averaged over those who are being referred), $\bar{p^{*}}=\sum_{i=1}^{N}p_{i}\bar{k}\left(p_{i}\right)/\left(NM\right)$, was a decreasing function of the rewiring threshold. This implies that the more the agents seek better referents, the lower is the mean ability of referents. These apparently counterintuitive results are discussed in Section 4.

The group performance and the performance of each agent also differed by the threshold. The mean group performance and the performance of each agent became better as the threshold *θ* decreased (Figs 8(a) and 9). The SD of the group performance, i.e., the fluctuation of the group performance, also increased as the threshold *θ* decreased (Fig 8(b)).

Therefore, when we set the threshold lower, the heterogeneity in in-degrees became stronger, and reference links were biased more toward higher ability agents. At the same time, we also see that the group performance became better on average, though its temporal fluctuation became greater. We discuss the reason why these results hold in the following Section 4.

## 4 Discussion

In this paper, we have shown that the reference structure of agents who try to make correct answers by referring to credible agents self-organized into a heterogeneous structure with an exponential in-degree distribution [26]. The mean in-degree increased exponentially with ability. Therefore small difference in ability can lead to large difference in the number of followers in the evolved network. Our analytical calculation shows that it was the mean duration of an agent to be kept linked by a follower that increased exponentially with his/her performance. The performance-monitoring process in our model generated this nonlinear relationship between performance and mean duration.

We also looked at the performance of each agent and that of the group in the evolved network and compared them to those in the random network. The mean performance of each agent and the mean group performance improved in the evolved network through adaptive rewiring compared with the random network. However, the fluctuation of the group performance in the evolved network was larger than the one in the random network. We discuss this trade-off later in this section.

In addition, we found that the threshold for rewiring, that is the extent of severity, affected the strength of heterogeneity in the in-degrees in the evolved network. When we set the threshold lower, the heterogeneity in the in-degrees became larger, and at the same time, the dependence of an agent’s mean in-degree on his/her ability was more exaggerated, i.e., agents refer more to higher ability agents in the evolved network, and the mean ability of referents increases. This leads to a higher mean performance of each agent compared with when the threshold was larger, i.e., when the mean ability of referents was lower. Actually, in our derivation of the mean performance explained in Section 3.2 and Section A in S1 Text, which predicts the simulation result well, we can show that the mean performance of each agent is an increasing function of the mean ability of referents (Section B in S1 Text). However, it is a little against our intuition that agents result in referring to higher ability agents when we set the threshold lower (i.e., when they were more generous to their referents) than when we set it higher (when they were stricter regarding their referents). We interpret this counterintuitive phenomenon as follows. A lower rewiring threshold makes each agent more patient and lowers the desire to kick-off low-ability referents. However, at the same time, a lower threshold contributes to keeping high-ability referents more securely, because a lower rewiring threshold leads to a longer duration for referent-monitoring, leading to a better overall sorting of referent’s quality. From our computer simulations, we find that the later effect is stronger. Therefore, in our model, a lower rewiring threshold contributes to generating a more biased reference toward high-ability agents. This result in our study can be tested by an empirical study comparing the generosity of societies and their accuracy in decision-making. For example, we may compare a group in which rewiring occurs easily (that may correspond to a high threshold in our model) such as a group of individuals connected by a social network service, with a group in which rewiring is difficult (that may correspond to a low threshold in our model) such as a group of individuals in a company who are connected tightly, to examine which group can predict the next political leader more accurately.

As we showed so far, how long one can keep a follower greatly affects the structure of the evolved network. The extent to which people in the society attach importance to the current result as compared to the referent’s past is measured by the parameter *α*. Its reciprocal, 1/*α*, gives the mean time an individual remembers a success or a failure of its referent. Indeed, the change in the evaluated performance of the referent, $y_{t}^{ij}$, per each time step is proportional to *α*: $\Delta y_{t}^{ij}=y_{t+1}^{ij}-y_{t}^{ij}=\alpha \left(I_{t+1}^{j}-y_{t}^{ij}\right)$. In the numerical simulations of this paper, we set *α* = 0.1. As *α* becomes larger, the agent’s evaluation becomes less dependent on the past and more heavily dependent on the immediate success or failure. This makes the evaluation of followers’ performance less reliable. Therefore, a larger *α* makes it difficult to sort subtle difference in performance between the referents, resulting in weaker centralization of links toward high ability agents and low performance. The effect of the kick-off threshold *θ* on group performance would also become less pronounced because of the less reliable performance evaluation. Conversely, if *α* becomes smaller, the evaluation for the performance of referents would become more reliable. However, this raises another problem for a society, because the time required for the referent network to reach an equilibrium, in other words, to acquire high centralization, would become too long. In fact, we confirmed those predictions on the effect of *α* by computer simulations for several values of *α*. The results are shown in S2 Text.

There are several trade-offs in our model that affect the understanding of the quality of decision-making by agents who are interacting with one another. First, when we set a lower rewiring threshold, we have to wait longer until the network reaches the equilibrium state where agents have higher mean performance. Thus, we can see a kind of *speed–accuracy trade-off* here. Second, along with stepwise rises of the group performance from independent decision, to random references in the initial state, and then to the high-ability-agent-oriented evolved networks, the SD of the group performance also increased, i.e., the fluctuation became larger in this order. When we set the threshold lower, we saw again an increase in both the mean and the SD of the group performance in the evolved network. Therefore, an increase in both the mean and the “stability” (suppression of fluctuation) are difficult to be compatible. High-ability agents collect more followers in the evolved network than in the initial network; the same is true for the evolved network of a low threshold compared with that with a high threshold. Adaptive rewiring and a lower kick-off threshold level lead to higher mean performance. However, this is due to a more intense concentration of reference links to high ability agents (Section B in S1 Text). This polarization seems to be the reason for the larger fluctuation of the group performance. The agents who attract many followers tend to be the agents with high ability and high performance. However, there are of course cases in which high-ability agents give wrong answers. In such an occasion of failure by agents of high influence, the group performance results in a very low value, which results in the group performance fluctuating wildly.

We have examined only a few types of distribution of agent’s ability in the population, which gives the seeds for the generation of a heterogeneous in-degree distribution through adaptive rewiring. Actually, we assumed two types of ability distributions—one is in the current study, the uniform distribution, and the other is shown in Section E in S1 Text. The density distribution of ability shown in Section E in S1 Text is a linear decreasing function on the interval [0.5, 0.75]. Although both forms of ability distribution yielded exponential in-degree distributions against varying ability, the robustness of the results for the other forms of ability distributions should be tested in the future.

Lastly, we discuss possible modifications of our model. In our model, we assumed that a new link comes randomly regardless of his/her ability value. This was based on the idea that one cannot know the status of strangers —this may be true in some cases in our society. For example, in a population of analysts where a lead-follow relationship (references) exists, a financial analyst may not be able to evaluate the correctness of the analysts whom he/she is not directly following. In such situations, the only thing that an agent can do to improve his/her own performance is to replace an already connected referent who did not give correct answers, with a new referent randomly chosen from the population [27] as we assumed in our model. Actually, an empirical work on a social network in a university [27] shows that such global rewiring is commonly found in a group of individuals sharing the same interaction focus (in our case, making decisions for the same problem). However, it may also be possible to introduce “reputation” into our model; i.e., we may assume that the probability of being newly chosen as a referent depends on one’s ability or performance, which is recognized by others in some way such as via reputation. We predict that, under this assumption, we will obtain a scale-free network, which represents strong heterogeneity. Several reasons support this prediction. There are a number of studies that explain how scale-free networks are constructed. The “good get richer” mechanism (or fitness model) is one such explanation [23, 24, 28]. In the models using the “good get richer” mechanism, each agent is assigned a value, such as *fitness*, and the probability that one can obtain a link is determined based on the *fitness* value. In these models, strong heterogeneity with a power-law degree distribution emerges even if the fitness is not power-law distributed. The *fitness* in such models corresponds to the ability component in our model. Thus, we can predict that we will obtain a scale-free network if the probability of being newly chosen depends directly on one’s ability or on one’s performance. It is not clear whether a population can achieve high performance under a structure that evolved in the presence of “reputation” and whether it has high heterogeneity and/or a strong opinion correlation. In addition, we think that the following issue is worth considering in future. In our study, we assumed that all agents follow the same strategy for decision-making and have the same rewiring threshold. With these simple assumptions, we were able to reveal what is the primarily factor leading to the centralization of reference networks, and to discuss the decision accuracy in the self-organized reference structure. A possible next step would be to analyze the model that allows ability-dependent strategy for each agent, as higher ability agents may have less motivation for referring to others than lower ability agents. If so, the presence of such independent decision makers would improve the efficiency of collective intelligence in the population [29].

## Supporting information

S1 Text Detailed explanation about analytical and numerical calculation in the main text. (PDF)

S2 Text The effect of the parameter *α* that represents the extent to which an agent attaches importance to the immediate past result in evaluating the performance of referents. (PDF)
