# Supplementary material for: Emergence of opinion leaders in reference networks
Source: PLoS One. 2018 Mar 26;13(3):e0193983. doi: 10.1371/journal.pone.0193983 (PMC5868794; doi:10.1371/journal.pone.0193983)
Supplement: S1 Text — (PDF) [file pone.0193983.s001.pdf]

S1. Supporting information for Emergence of opinion leaders in reference networks

A The derivation of the mean performance

Derivation of mean performance

The mean performance of an agent can be described as a linear function of his/her ability as we explained in Section 3.2 in the main text. Here we explain the derivation of the approximate formula for the slope and the intercept of the linear function. In this derivation, we neglected self-loops or overlaps in reference links to simplify our approximation.

In our model, agents make their decisions sequentially, so each agent actually incorporates answers of only referents who have already stated the answer (let us call them "stated referents") among M agents to which he/she links (out-degree$\equiv M$). Therefore, in sequential decision-making, agents are divided into $M+1$ classes: class C_0 of agents who make decisions independently by themselves, class C_1 of agents who put the answer of a referent together with his/her own choice, and so on. Let C_s be the class of agents who put the answers of s referents together with their own choice. There are on average

$$c_s = \sum_{l=s+1}^{N} \binom{M}{s} \left(\frac{l-1}{N}\right)^s \left(1 - \frac{l-1}{N}\right)^{M-s} \tag{S.1}$$

agents in such class as shown below. Suppose that the agent is the l-th earliest to make the decision (l can be chosen from $s+1$ to N with equal probability). Given that the agent is the l-th earliest, there are $l-1$ agents who have already stated their answers. Thus, the number of stated referents of the l-th earliest agent follows the binomial distribution with parameters M and $(l-1)/N$, since agents state their answers in a randomly determined order. This leads to the expression (S.1) for the mean number of agents who belong to class C_s.

To derive an approximation formula for the mean performance, we assumed that all the agents who made their decisions the earliest to the c_0-th earliest belong to class C_0, i.e., they had no stated referent in making their decision; hence, they decided relying only on their own belief. Similarly, all

the agents who made the $(c_{s-1}+1)$-th earliest to the c_s-th earliest are assumed to belong to class C_s, $(s = 1, 2, \ldots, M)$, i.e., put the answers of s referents together with their own choices in making their decisions. Here, we further assume that s referents of an agent of class C_s are randomly chosen from the agents of class C_0, C_1, …, C_{s-1} in proportion to $c_0 : c_1 : \cdots : c_{s-1}$.

Let $\pi_s(p)$ be the mean performance of an agent who has ability p and belongs to class C_s. Clearly, $\pi_0(p) = p$. Let r_s^* be a random variable that represents the performance of a referent who is being referred by the agents in class C_s. Let us consider an agent who is randomly sampled from the population, say agent i. We approximated the probability that a referent of agent i has ability p as $g(p) \equiv \bar{k}(p)/(NM)$ regardless of the number of followers of the agent, where $\bar{k}(p)$ is the mean in-degree of the agent with ability p, as explained in Section 3.1 in the main text (Eq.(2)). Under this assumption, let p^* be a random variable that represents a referent's ability. Thus, we assumed that the ability of a referent p^* follows the distribution g. Using the above simplifying assumptions, r_1^* can be approximated as $r_1^* \approx \pi_0(p^*) = p^*$ since r_1^* is the performance of an agent in class C_0. Furthermore, we approximated the value of $r_1^* = p^*$ by its mean, $\overline{p^*} \equiv \mathrm{E}[p^*] \approx \sum_{i=1}^{N} p_i g(p_i) = \sum_{i=1}^{N} p_i \bar{k}(p_i)/(NM)$.

The performance of an agent in class C_1 can be described as

$$\pi_1(p) = p r_1^* + \frac{1}{2}\left[p(1-r_1^*) + (1-p)r_1^*\right] \tag{S.2}$$

$$= \frac{p + r_1^*}{2}. \tag{S.2$'$}$$

The first term on the right-hand side of (S.2) stands for the contribution to the mean performance when both the referent and the agent him/herself gave correct answers. The second term is the contribution when either the referent or the agent him/herself gave a correct answer (the two different opinions tie in this case, and the actual decision is made by tossing a coin; hence, the factor 1/2). Similarly, $\pi_1(p)$ can be approximated as follows:

$$\pi_1(p) = \frac{p + r_1^*}{2} \approx \frac{p + p^*}{2}$$

$$\approx \frac{p + \overline{p^*}}{2}. \tag{S.3}$$

Now, we consider the mean performance of referents who are being referred by the agents in class C_2, $r_2 \equiv \overline{r_2^*}$, as follows. Under our assumption, an agent in class C_2 refers to an agent in class C_0 with a probability of $c_0/(c_0 + c_1)$ and refers to an agent in class C_1 with a probability of $c_1/(c_0 + c_1)$. Thus,

$$r_2^* = \begin{cases} \pi_0(p^*), \text{ with probability } \dfrac{c_0}{c_0 + c_1}, \\ \pi_1(p^*), \text{ with probability } \dfrac{c_1}{c_0 + c_1}. \end{cases} \tag{S.4}$$

Since $\pi_0(p) = p$, and by equation (S.3), functions π_0 and π_1 can be assumed as linear functions—we can see that $\pi_0(p)$ and $\pi_1(p)$ also increase nearly linearly with ability p in our computer simulation (Fig.S.1). Therefore, the mean performance of referents who are being referred by the agents in class C_2, r_2, can be approximated as

$$\begin{aligned} r_2 &\approx \mathrm{E}\left[\frac{c_0}{c_0 + c_1}\pi_0(p^*) + \frac{c_1}{c_0 + c_1}\pi_1(p^*)\right] \\ &= \frac{c_0}{c_0 + c_1}\mathrm{E}[\pi_0(p^*)] + \frac{c_1}{c_0 + c_1}\mathrm{E}[\pi_1(p^*)] \\ &\approx \frac{c_0}{c_0 + c_1}\pi_0(\overline{p^*}) + \frac{c_1}{c_0 + c_1}\pi_1(\overline{p^*}), \end{aligned} \tag{S.5}$$

where $\pi_0(p) = p$ and $\pi_1(p) = (p + \overline{p^*})/2$. Therefore,

$$r_2 \approx \frac{c_0}{c_0 + c_1}\overline{p^*} + \frac{c_1}{c_0 + c_1}\frac{\overline{p^*} + \overline{p^*}}{2} = \overline{p^*}. \tag{S.6}$$

Similarly, $\pi_2(p)$, r_3, $\pi_3(p)$, …, r_M, and $\pi_M(p)$ can be derived sequentially as follows: Let $Q(s, j)$ be the probability that j out of s stated referents of an agent in class C_s give correct answers, as

$$Q(s, j) = \binom{s}{j} r_s^j (1 - r_s)^{s-j}. \tag{S.7}$$

When the number of stated referents of an agent is even, there are $s + 1$ answers/choice including his/her own choice. The agent can give a correct answer by majority-rule when more than $s/2$ answers/choice are correct. Therefore, the mean performance of an agent with ability p in class C_s,

$\pi_s(p)$, can be described as

$$\begin{aligned}\pi_s(p) &= p \sum_{j+1 \geq s/2+1} Q(s,j) + (1-p) \sum_{j \geq s/2+1} Q(s,j) \\ &= p \sum_{j=s/2}^{s} Q(s,j) + (1-p) \sum_{j=s/2+1}^{s} Q(s,j) \\ &= \sum_{j=s/2+1}^{s} Q(s,j) + pQ\left(s, \frac{s}{2}\right), \end{aligned} \tag{S.8}$$

when s is even. Similarly, when s is odd,

$$\begin{aligned}\pi_s(p) &= p \sum_{j+1 \geq (s+1)/2+1} Q(s,j) + (1-p) \sum_{j \geq (s+1)/2+1} Q(s,j) \\ &\quad + \frac{1}{2}\left[pQ(s, \frac{s-1}{2}) + (1-p)Q(s, \frac{s+1}{2})\right] \\ &= \sum_{j=(s+3)/2}^{s} Q(s,j) + \frac{1}{2}Q(s, \frac{s+1}{2}) + \frac{1}{2}\left[Q(s, \frac{s-1}{2}) + Q(s, \frac{s+1}{2})\right] p. \end{aligned} \tag{S.9}$$

The mean performance of referents who are being referred by the agents in class C_s, r_s, can be derived as

$$\begin{aligned} r_s &= \frac{\sum_{j=1}^{s-2} c_j}{\sum_{j=1}^{s-1} c_j} r_{s-1} + \frac{c_{s-1}}{\sum_{j=1}^{s-1} c_j} \mathrm{E}[\pi_{s-1}(p^*)] \\ &= \frac{\sum_{j=1}^{s-2} c_j}{\sum_{j=1}^{s-1} c_j} r_{s-1} + \frac{c_{s-1}}{\sum_{j=1}^{s-1} c_j} \pi_{s-1}(\overline{p^*}). \end{aligned} \tag{S.10}$$

In equation (S.10), $\mathrm{E}[\pi_{s-1}(p^*)] = \pi_{s-1}(\mathrm{E}[p^*]) = \pi_{s-1}(\overline{p^*})$ holds because $\pi_{s-1}(p)$ can be inductively assumed to be a linear function of p for any s according to Equations (S.7)–(S.10), and from Fig. S.1—r_s is not a function of p but a function of $\overline{p^*} = \mathrm{E}[p^*] \approx \sum_{i=1}^{N} p_i g(p_i)$. Therefore, the mean performance of agents in class C_s can be represented as

$$\pi_s(p) = A_{g,s} p + B_{g,s}, \tag{S.11}$$

where $A_{g,s}$ and $B_{g,s}$ do not depend on p.

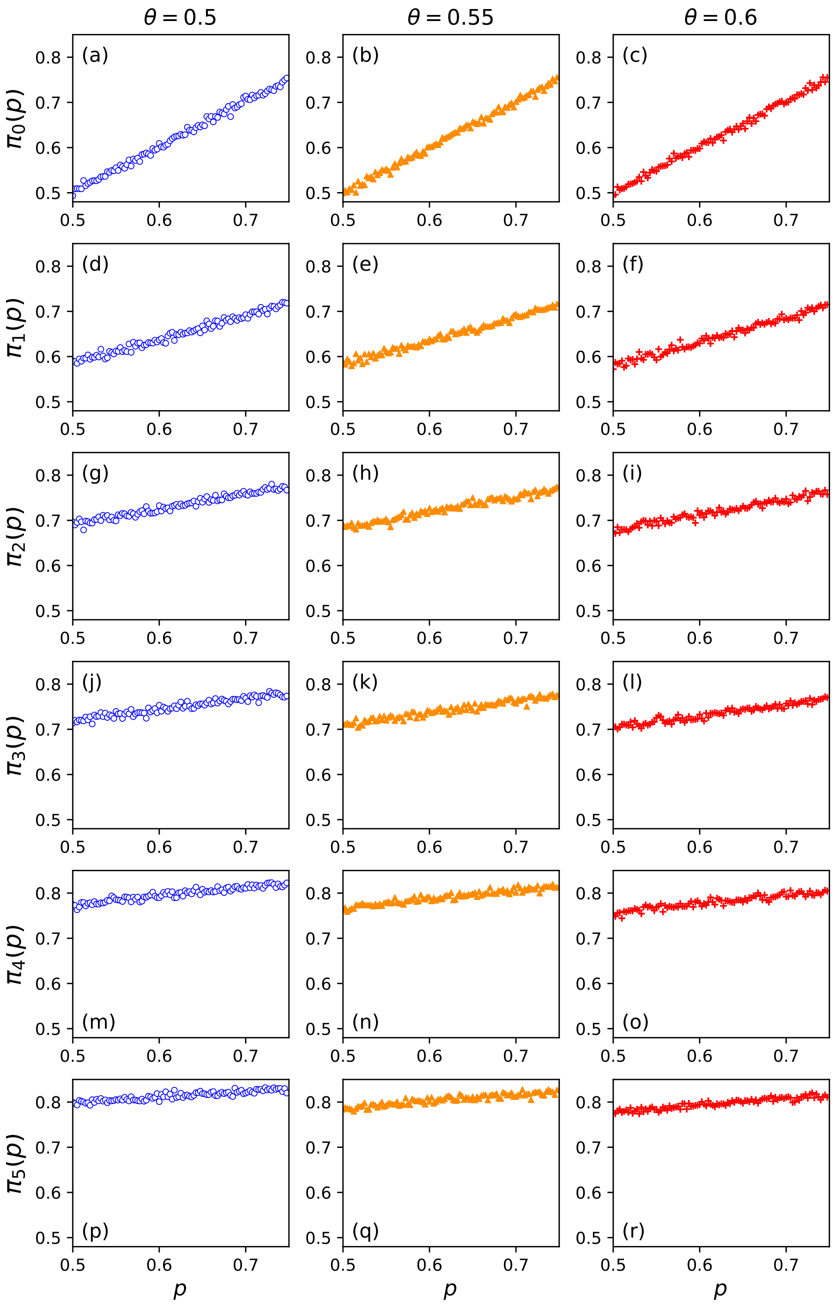

Figure S.1: The mean performance of an agent in class C_s, $\pi_s(p)$ versus his/her ability p are shown for $s = 0, 1, \ldots, 5$ and for rewiring thresholds $\theta = 0.5$, 0.55 and 0.6. Figures in the first, second, third, forth, fifth and sixth rows show the case where the number of stated referents s are 0, 1, 2, 3, 4 and 5, respectively. Figures in the first, second and third columns show the case where the threshold θ are 0.5, 0.55 and 0.6, respectively. In each panel, we can see the linear increase of the mean performance with the ability.

The mean performance depends linearly on the agent's ability.

Finally, the mean performance $\pi(p)$ of an agent with ability p can be approximated as

$$\pi(p) = \frac{\sum_{s=0}^{M} c_s \pi_s(p)}{\sum_{s=0}^{M} c_s}. \tag{S.12}$$

Since $\pi_{s-1}(p)$ is a linear function of p for each s, $\pi(p)$ is also a linear function of p and can be represented as

$$\pi(p) = A_g p + B_g. \tag{S.13}$$

The mean performance calculated by equation (S.13) agrees with the simulation results shown in Fig. 5 (a).

The linearity of mean performance when self-loops and overlaps are allowed

Here, we show that the mean performance can be described as a linear function of the ability even if there are self-loops and overlaps in reference links. First, we consider the case where there are $a_{ii}(\geq 1)$ self-loops of an agent i, where we regard that agent i's choice has a weight of $(a_{ii}+1)$ to him/herself in i's majority-rule voting. We can calculate the mean performance of agent i as follows. Let s be a_{ii} plus the number of the agent i's stated referents other than agent i. Let $\{d_0, d_1, \ldots, d_{a_{ii}}, \ldots, d_s\}$ be a set of choices and answers, where d_0 represents the choice of agent i him/herself and each of $d_1, \ldots, d_{a_{ii}}$ represents the choice of agent i him/herself relating to the a_{ii} self-loops ($d_1 = d_2 = \cdots = d_{a_{ii}} = d_0$ by definition). Each of $d_{a_{ii}+1}, \ldots, d_s$ represents the answer of each stated referent of agent i. In this case, $(d_0, d_1, \ldots, d_{a_{ii}}) = (1, 1, \ldots, 1)$ with a probability of p_i and $(d_0, d_1, \ldots, d_{a_{ii}}) = (0, 0, \ldots, 0)$ with a probability of $1-p_i$. From equation (S.8), the probability

that agent i makes a correct answer, $\pi_s(p_i)$, is,

$$\begin{aligned}\pi_s(p_i) = \quad & p_i\text{Pr.}\Bigg[\left(\frac{(s+1)+1}{2} - (a_{ii}+1)\right)\text{stated referents} \\ & \qquad\qquad \text{other than agent } i \text{ gave correct answers}\Bigg] \\ & +(1-p_i)\text{Pr.}\Bigg[\left(\frac{(s+1)+1}{2}\right)\text{stated referents} \\ & \qquad\qquad\qquad \text{other than agent } i \text{ gave correct answers}\Bigg], \qquad \text{(S.14)}\end{aligned}$$

when s is even. Note that $\pi_s(p_i)$ shown above is a linear function of p_i. Using the case that s is odd in Equation (S.9), we can again describe $\pi_s(p_i)$ as a linear function of p_i when s is odd. Similarly, we can confirm that the mean performance of an agent can be described as a linear function of the ability even when there are overlaps in reference links.

B The relationship between the mean performance and the mean ability of referents

In Section A, we derived the approximation formula for the mean performance $\pi(p)$ of an agent with ability p, which is expressed in terms of the mean ability of referents $\overline{p^*} = \sum_{i=1}^{N} p_i \bar{k}(p_i)/(NM)$.

As discussed in the main text, adaptive rewiring and a lower kick-off threshold lead to both a high mean ability of referents $\overline{p^*}$ (FIG. 12) and a high mean performance of each agent (FIG. 9). In this section, we show that the mean performance of each agent increases with the mean ability of referents according to the formula for the mean performance that we obtained in Section A. We can, therefore, say that adaptive rewiring and a lower kick-off increase the mean ability of referents, and this leads to a high mean performance of each agent.

We will show that $\partial\pi(p)/\partial\overline{p^*} > 0$ for all p and $\overline{p^*}$ with $0 \le p \le 1$ and $0 \le \overline{p^*} \le 1$.

Proof. From the Equation (S.12) in Section A,

$$\frac{\partial\pi(p)}{\partial\overline{p^*}} = \frac{\sum_{i=0}^{M} c_s \partial\pi_s(p)/\partial\overline{p^*}}{\sum_{s=0}^{M} c_s}. \tag{S.15}$$

We show below that $\partial\pi_0(p)/\partial\overline{p^*} = 0$ and $\partial\pi_s(p)/\partial\overline{p^*} > 0$ for $s \ge 1$. Clearly $\partial\pi_0(p)/\partial\overline{p^*} = 0$ as $\pi_0(p) = p$, and $\partial\pi_1(p)/\partial\overline{p^*} = 1/2 > 0$ since $\pi_1(p) = (\overline{p^*} + p)/2$. For $s \ge 2$, we show both $\partial r_s/\partial\overline{p^*} > 0$ and $\partial\pi_s/\partial r_s > 0$ since $\partial\pi_s(p)/\partial\overline{p^*} = (\partial r_s/\partial\overline{p^*})(\partial\pi_s/\partial r_s)$.

First, we show $\partial\pi_s/\partial r_s > 0$. When s is even and $s \ge 2$, by equations (S.8) and (S.15),

$$\frac{\partial\pi(p)}{\partial\overline{p^*}} = \sum_{j=s/2+1}^{s} \frac{\partial Q(s,j)}{\partial r_s} + p\frac{\partial Q\left(s, s/2\right)}{\partial r_s}, \tag{S.16}$$

where $\partial Q(s,j)/\partial r_s = \binom{s}{j}[j r_s^{j-1}(1-r_s)^{s-j} - (s-j) r_s^{j}(1-r_s)^{s-j-1}]$. Using the fact that $\binom{m}{n}(m -$

$n) - \binom{m}{n+1}(n+1) = 0$ for any integers m and n with $m > n \geq 0$,

$$\frac{\partial \pi(p)}{\partial \overline{p^*}} = r_s^{s/2-1}(1-r_s)^{s/2-1} \times \left[\binom{s}{s/2-1}\left(\frac{s}{2}+1\right) r_s + p\binom{s}{s/2}\frac{s}{2}(1-r_s) - p\binom{s}{s/2}\frac{s}{2}r_s\right] \tag{S.17}$$

$$= r_s^{s/2-1}(1-r_s)^{s/2-1}\frac{s!}{(s/2-1)!(s/2)!}\left[(1-r_s)p + r_s(1-p)\right] > 0. \tag{S.17$'$}$$

Similarly, when s is odd and $s \geq 3$,

$$\frac{\partial \pi_s(p)}{\partial r_s} = \frac{1}{2}\binom{s}{(s-1)/2}\frac{s+1}{2}r_s^{(s-1)/2}(1-r_s)^{(s-1)/2} + \frac{1}{2}\frac{s!}{[(s-3)/2]![(s+1)/2]!}r_s^{(s-3)/2}(1-r_s)^{(s-3)/2}\left[(r_s-p)^2 + p(1-p)\right] > 0. \tag{S.18}$$

Therefore, $\partial \pi_s / \partial r_s > 0$ for $s \geq 2$.

Secondly, we show $\partial r_s / \partial \overline{p^*} > 0$ for $s \geq 2$. By equation (S.10) and (S.11),

$$\frac{\partial r_s}{\partial \overline{p^*}} = \frac{\sum_{j=1}^{s-2} c_j}{\sum_{j=1}^{s-1} c_j}\frac{\partial r_{s-1}}{\partial \overline{p^*}} + \frac{c_{s-1}}{\sum_{j=1}^{s-1} c_j}A_{g,s}. \tag{S.19}$$

Since $r_1 = r_2 = \overline{p^*}$, as explained in Section A, $\partial r_1 / \partial \overline{p^*} = \partial r_2 / \partial \overline{p^*} = 1 > 0$. Thus, inductively, $\partial r_s / \partial \overline{p^*} > 0$ for $s \geq 2$, according to Equation (S.19). □

C The formal derivation of the mean in-degree

Here, we assume that the number of agents N is infinitely large, and p is a continuous value. Let $\tilde{g}(p)$ be the probability density function for the ability of an agent who is being referred to in the evolved network. In addition, we define $f(\Pi)$ as the probability density function of the performance of the agent who is being referred to in the evolved network. $\psi(p)$ and $\phi(\Pi)$ respectively denote the unconditional probability density functions of the ability and performance of the agents, who are either being referred to or not. Let $\overline{T_\Pi}$ be the mean duration that the agent with performance Π is kept linked by a follower.

Let $f_t(\Pi)$ be the probability density function for the performance of the agent who is being referred to at iteration time t. The function $f_t(\Pi)$ satisfies the following equation by assuming that a link directing to an agent with performance Π is detached with a probability of $1/\overline{T_\Pi}$ in a unit time interval:

$$f_{t+1}(\Pi) = \left(1 - \frac{1}{\bar{T}_\Pi}\right) f_t(\Pi) + \phi(\Pi) \int_0^1 f_t(\Pi') \frac{1}{\bar{T}_{\Pi'}} d\Pi'. \tag{S.20}$$

The first term on the right-hand side of equation (S.20) corresponds to the probability that a reference link to an agent with performance Π remains without being rewired in a unit time interval. The second term corresponds to the probability that a link is newly rewired to an agent with performance Π after it is discarded. Therefore, in the equilibrium state, the probability density function for the performance of the agent who is being referred to in the evolved network, $f(\Pi)$, holds:

$$f(\Pi) = \left(1 - \frac{1}{\bar{T}_\Pi}\right) f(\Pi) + \phi(\Pi) \int_0^1 f(\Pi') \frac{1}{\bar{T}_{\Pi'}} d\Pi'. \tag{S.21}$$

Equation (S.21) can be calculated as follows.

$$0 = -\frac{f(\Pi)}{\bar{T}_\Pi} + \phi(\Pi) \int_0^1 \frac{f(\Pi')}{\bar{T}_{\Pi'}} d\Pi',$$

and hence,

$$f(\Pi) = \frac{\bar{T}_\Pi \phi(\Pi)}{\int_0^1 \bar{T}_{\Pi'} \phi(\Pi') d\Pi'}. \tag{S.22}$$

Since $\Pi(p) = A_g p + B_g$ from Equation (S.13) in Section A, $f(\Pi) = \tilde{g}((\Pi - B_g)/A_g)/A_g$ and

$\phi(\Pi) = \psi((\Pi - B_g)/A_g)/A_g$ are satisfied. Therefore,

$$\tilde{g}(p) = \frac{\bar{T}_{Ap+B}\psi(p)}{\int_0^1 \bar{T}_{Ap'+B}\psi(p')dp'}. \tag{S.23}$$

In the main text, we set $\psi(p)$ as

$$\psi(p) = \begin{cases} 4, & 0.5 \leq p \leq 0.75, \\ 0, & \text{otherwise.} \end{cases} \tag{S.24}$$

i.e., p follows the uniform distribution U(0.5, 0.75). For the finite number of agents N, we can approximate the probability $g(p_i)$ that agent i with ability p_i is being referred from an agent as $g(p_i) \approx \int_{p_i}^{p_{i+1}} \tilde{g}(p')dp'$. Thus the solid lines in FIG. 7 (b) are calculated as

$$\begin{aligned} \bar{k}(p_i) =& NMg(p_i) \\ \approx& NM \int_{p_i}^{p_{i+1}} \tilde{g}(p')dp' = NM \int_{p_i}^{p_{i+1}} \frac{\bar{T}_{Ap+B}}{\int_0^1 \bar{T}_{Ap'+B}dp'}dp \\ \approx& NM \frac{\bar{T}_{Ap_i+B}}{\sum_{j=1}^{N} \bar{T}_{Ap_j+B}}. \end{aligned} \tag{S.25}$$

D The numerical procedure to obtain the mean duration that an agent keeps a follower

In this section, we explain how we solve the recurrence equations (5) and (6) in Section 3.3 in the main text,

$$T_\Pi(y) = 1 + \Pi T_\Pi(\alpha + (1-\alpha)y) + (1-\Pi)T_\Pi((1-\alpha)y), \quad y > \theta, \tag{S.26}$$

and

$$T_\Pi(y) = 0, \quad y \leq \theta, \tag{S.27}$$

for the recurrence of $T_\Pi(y)$. Here $T_\Pi(y)$ represents the mean time until the evaluated performance Y_t of a referent, whose actual performance is Π, hits the threshold θ first time in the stochastic process $\{Y_t \mid Y_0 = y\}$, where t is the time since it is linked by a follower.

Note that the evaluated performance Y_t is always less than 1, because the right side of (1) in the main text represents the internally dividing point of I_t, which is either 0 or 1, and the current value of Y_t. Let b be $(1-\alpha)\theta$, which is the infimum of the realization of Y_t because the evaluated performance is updated to $(1-\alpha)\theta$ when a referent with its evaluated performance θ gives a wrong answer. We discretized the interval $[b, 1] (\subset \mathbb{R})$ as $B \equiv \{b_0, b_1, \ldots, b_S\}$, where $b_i = b + i\delta$ and $S = [(1-b)/\delta]$, and consider the recurrence equations (S.26) and (S.27) on B, as follows. Here we set δ sufficiently small as $\delta = 0.0001$ in our actual numerical calculation and $[\,\cdot\,]$ represents the Gauss' symbol.

We defined four maps, $\mathcal{I}$, $\mathcal{R}$, $\mathcal{U}$ and $\mathcal{D}$ as follows.

$$\mathcal{I}(y) \equiv [(y-b)/\delta], \tag{S.28}$$

with which $i = \mathcal{I}(b_i)$, representing the discretization of the interval $[b, 1]$, and its inverse

$$\mathcal{R}(j) \equiv b + \delta j, \tag{S.29}$$

with which $b_i = \mathcal{R}(i)$,

$$\mathcal{U}(y) \equiv (1-\alpha)y + \alpha, \tag{S.30}$$

and

$$\mathcal{D}(y) \equiv (1-\alpha)y. \tag{S.31}$$

The map $\mathcal{U}$ ($\mathcal{D}$) corresponds to the case that the referent gives a correct (wrong) answer and the evaluated performance Y_t changes better (worse). Therefore, the function $\mathcal{I} \circ \mathcal{U} \circ \mathcal{R}$ ($\mathcal{I} \circ \mathcal{D} \circ \mathcal{R}$) means the change in the discretized evaluated performance when the referent gives a correct (wrong) answer, where $\circ$ means composition of functions.

Let $\boldsymbol{x} = (x_i)$ be a $(S+1)$-dimensional vector, where $x_i = T_\Pi(b_i)$. With this definition, x_i gives the mean first hitting time when $Y_0 = y = b + i\delta$, to the threshold θ of the stochastic process $\{Y_t \mid Y_0 = b + i\delta\}$. The recurrence equations (S.26) and (S.27) for discretized state space in the evolved network are then expressed as

$$\boldsymbol{x} = A\boldsymbol{x} + \mathbf{1}, \tag{S.32}$$

where $A = (a_{ij})_{i,j=0,\ldots,S}$ is an $(S+1)\times(S+1)$ matrix where the first $\mathcal{I}(\theta)+1$ rows of A are zero vectors,

$$a_{ij} = 0, \qquad i = 0, 1, ..., \mathcal{I}(\theta);\ \ j = 0, 1, \ldots, S. \tag{S.33}$$

From the $i = \mathcal{I}(\theta)+1$st to $i = \mathcal{I}\big(\theta/(1-\alpha)\big)$th rows of A are vectors in which only $(i, \mathcal{I} \circ \mathcal{U} \circ \mathcal{R}(i))$-element is non zero value, Π,

$$a_{ij} = \begin{cases} \Pi, & i = \mathcal{I}(\theta)+1, \ldots, \mathcal{I}\big(\theta/(1-\alpha)\big),\ \ j = \mathcal{I} \circ \mathcal{U} \circ \mathcal{R}(i), \\ 0, & i = \mathcal{I}(\theta)+1, \ldots, \mathcal{I}\big(\theta/(1-\alpha)\big),\ \ j \neq \mathcal{I} \circ \mathcal{U} \circ \mathcal{R}(i). \end{cases} \tag{S.34}$$

From the $i = \mathcal{I}\big(\theta/(1-\alpha)\big)+1$st to Sth rows of A are vectors in which only $(i, \mathcal{I} \circ \mathcal{D} \circ \mathcal{R}(i))$-element

and $(i, \mathcal{I} \circ \mathcal{U} \circ \mathcal{R}(i))$-element are non zero values, $1 - \Pi$ and Π, respectively,

$$a_{ij} = \begin{cases} 1 - \Pi, & i = \mathcal{I}(\theta) + 1, \dots, S, \ \ j = \mathcal{I} \circ \mathcal{D} \circ \mathcal{R}(i), \\ \Pi, & i = \mathcal{I}(\theta) + 1, \dots, S, \ \ j = \mathcal{I} \circ \mathcal{U} \circ \mathcal{R}(i), \\ 0, & i = \mathcal{I}(\theta) + 1, \dots, S, \ \ j \neq \mathcal{I} \circ \mathcal{D} \circ \mathcal{R}(i), \mathcal{I} \circ \mathcal{U} \circ \mathcal{R}(i). \end{cases} \tag{S.35}$$

and $\mathbf{1} = (1_i)_{i=0,\dots,S}$ is an $(S+1)$-dimensional vector with

$$1_i = \begin{cases} 0, & i = 0, 1, \dots, \mathcal{I}(\theta), \\ 1, & i = \mathcal{I}(\theta) + 1, \dots, S. \end{cases} \tag{S.36}$$

By equation (S.27), the first $\mathcal{I}(\theta) + 1$ elements in the vector $\mathbf{1}$ are 0, and the first $\mathcal{I}(\theta) + 1$ rows in the matrix A are zero vectors. Since the third term $(1 - \Pi)T_\Pi((1 - \alpha)y)(= (1 - \Pi)T_\Pi(\mathcal{D}(y)))$ in the right side of equation (S.26) does not vanish when the initial evaluated performance y satisfies $(1 - \alpha)y > \theta$ (i.e. $y > \theta/(1 - \alpha)$), the $(i,\ \mathcal{I} \circ \mathcal{D} \circ \mathcal{R}(i))$-element of the matrix A is $1 - \Pi$ for $i > \mathcal{I}(\theta/(1 - \alpha))$. The second term $\Pi T_\Pi(\mathcal{U}(y))$ in the right side of equation (S.26) does not vanish when the initial evaluated performance y satisfies $y > \theta$. Thus the $(i,\ \mathcal{I} \circ \mathcal{U} \circ \mathcal{R}(i))$-element of the matrix A is Π for $i > \mathcal{I}(\theta)$.

The equation (S.32) can be solved as

$$\boldsymbol{x} = (E - A)^{-1}\mathbf{1}, \tag{S.37}$$

where E represents the identity matrix.

The $\mathcal{I}(y_0)$-th element in the vector $\boldsymbol{x}$ is the duration that an agent with the performance Π is kept linked from a follower, $\overline{T}$, for the initial evaluated performance $Y_0 = y_0$.

E Another distribution of ability

To check the validity of our way to derive $g(p)$, we apply it to another probability density function (p.d.f) of agent's ability. The applied p.d.f. has a saw-toothed shape with the vertical tip at $p = 0.5$, declining linearly with p towards zero at $p = 0.75$:

$$\tilde{\psi}(p) = \begin{cases} 24 - 32p, & 0.5 \leq p \leq 0.75, \\ 0, & \text{otherwise.} \end{cases} \tag{S.38}$$

We calculated the p.d.f. of the ability of being referred agents $\tilde{g}(p) = \tilde{\psi}(p)\bar{T}_{Ap+B} / \int_0^1 \tilde{\psi}(p')\bar{T}_{Ap'+B}dp'$ as the same way for $\psi(p)$. To compare with the calculation results, we conducted simulation under parameters $N = 100$, $M = 5$, $y_0 = 0.625$ and $\alpha = 0.1$. We set the ability of agents as $p_i = (3 - \sqrt{1 - i/N})/4$, $i = 0, 1, 2, \ldots$, since $a = (3 - \sqrt{1 - u})/4$ follows the equation (S.38), where u is a random variable which follows the uniform distribution in $[0, 1]$. The simulation data of the mean in-degree $\bar{k}(p_i)$ of an agent with the ability p_i agrees with $NM \int_{p_i}^{p_{i+1}} \tilde{g}(p')dp'$, see Fig. S.2. Note that we obtained the exponential tailed in-degree distribution again with this p.d.f. $\tilde{\psi}$ (Fig. S.3). As shown in Figure S.2, the mean in-degree also increases exponentially with the ability.

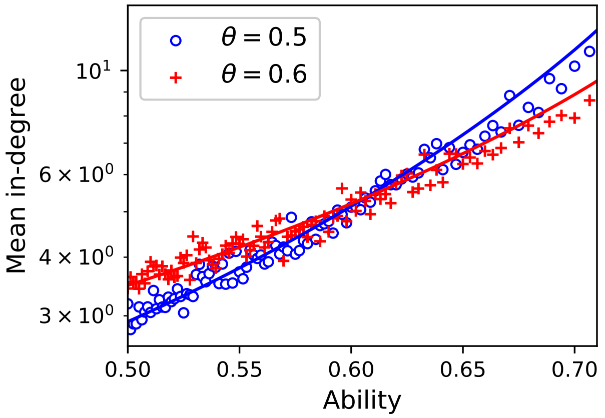

Figure S.2: The semi-log plot of the mean in-degree of an agent versus his/her ability in the evolved network. Different symbols represent results for 2 thresholds, $\theta = 0.5$ and 0.6. The distribution of the abilities is given by $\tilde{\psi}(p)$. The mean in-degree increases approximately exponentially with ability. The circle and plus are simulation data and the analytical results are shown by solid lines.

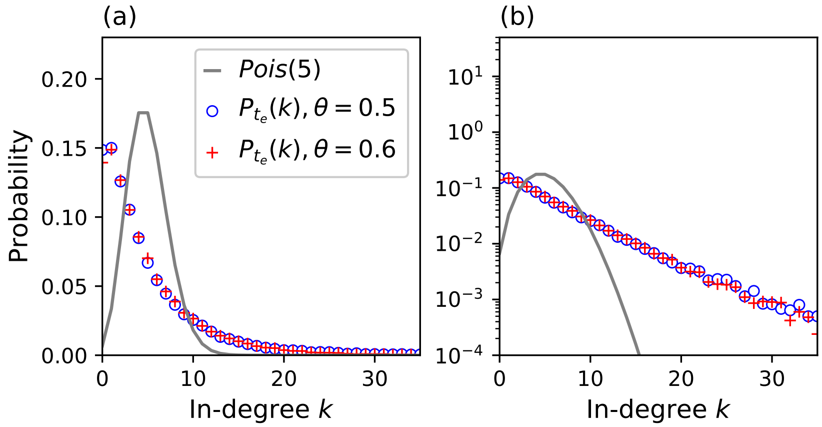

Figure S.3: (a) The solid line is the Poisson distribution with mean 5 representing the initial in-degree distribution. The markers (circle and plus) show the in-degree distributions of the evolved network with rewiring threshold $\theta = 0.5$ and 0.6, respectively, obtained by 500 independent runs of our simulation. (b) The same as (a) except that the vertical axis is logarithmically scaled. We can see approximately exponential tails in the evolved networks. In both of these figures, the distribution of the abilities is given by $\tilde{\psi}(p)$.
