# Supplementary material for: Emergence of opinion leaders in reference networks
Source: PLoS One. 2018 Mar 26;13(3):e0193983. doi: 10.1371/journal.pone.0193983 (PMC5868794; doi:10.1371/journal.pone.0193983)
Supplement: S2 Text — (PDF) [file pone.0193983.s002.pdf]

S2. Supporting information for Emergence of opinion leaders in reference networks

The effect of the parameter α

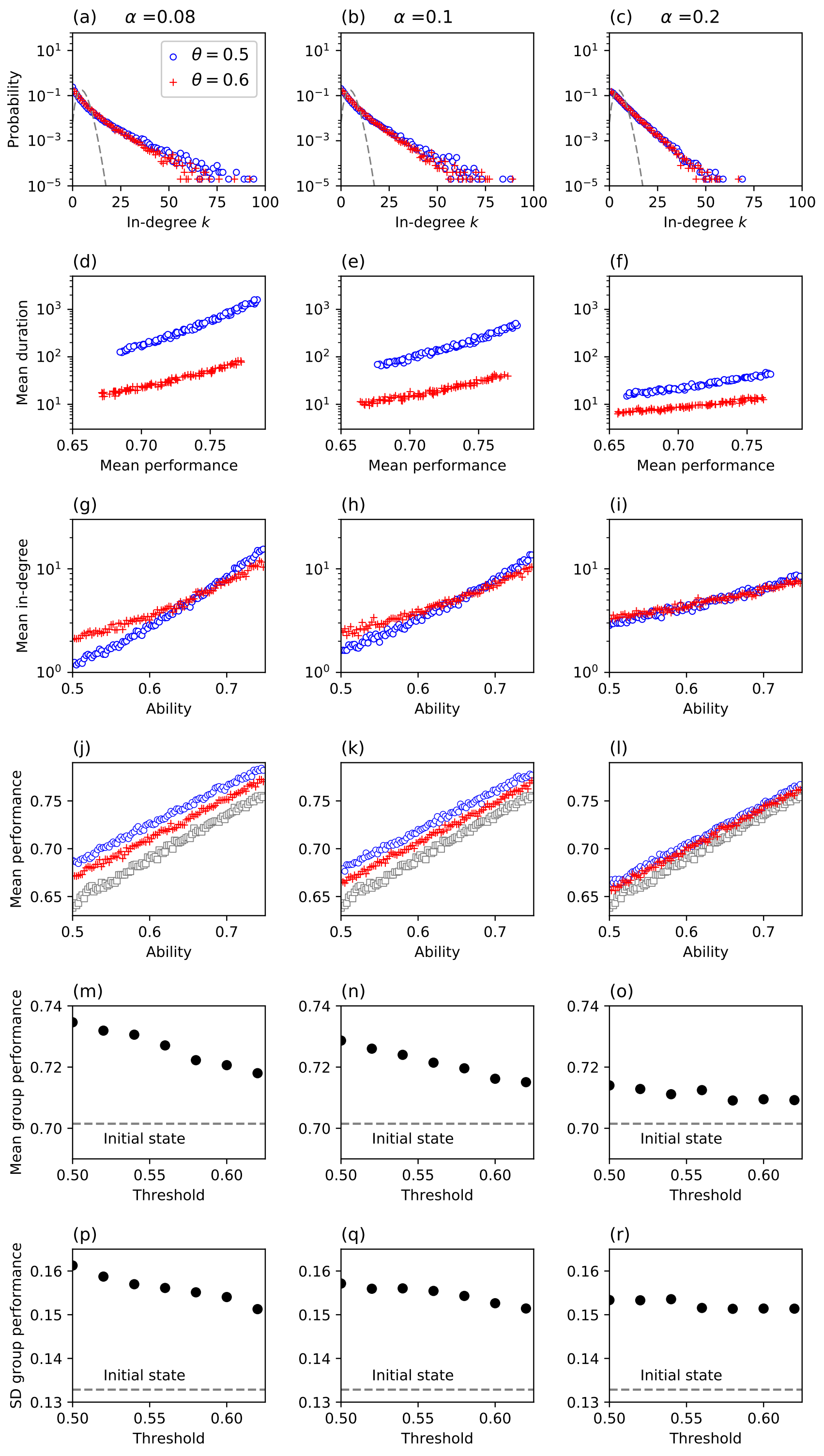

Figure S2.1: **The effect of the parameter α that represents the extent to which an agent attaches importance to the immediate past result in evaluating the performance of referents.** Panels in the first to the third columns respectively show the results for α = 0.08, 0.1 and 0.2. In all panels, the blue circles represent the results for the rewiring threshold θ = 0.5, and the red crosses are for θ =0.6, in the evolved networks.
(a)-(c): The in-degree distributions in the initial random network (dashed) and in the evolved networks with different rewiring thresholds θ =0.5 (blue circles) and θ =0.6 (red crosses). The vertical axis is scaled logarithmically. The initial in-degree distribution follows the Poisson distribution with mean 5. The tails in the in-degree distributions in the evolved networks are approximately exponential for all cases. The tails of in-degree distributions show steeper declines as α becomes larger.
(d)-(f): The mean duration that an agent is kept by a follower plotted against the agent's mean performance in the evolved networks with different rewiring thresholds θ =0.5 (blue circles) and θ =0.6 (red crosses). The mean duration increases approximately exponentially (but actually slightly faster than exponentially) with the mean performance for all α values. As α becomes larger, the mean duration of keeping a follower declines. Consequently, both the effects of the mean performance (the slopes of curves) and of the rewiring thresholds (the difference between blue and red points) on the mean duration become less pronounced.
(g)-(i) The mean in-degree of an agent plotted against his/her ability in the evolved networks with rewiring thresholds 0.5 (blue circles) and 0.6 (red crosses). The vertical axis is scaled logarithmically. The mean in-degree increases approximately exponentially (but actually slightly faster than exponentially) with ability for all values of α. The effect of the ability or the kick-off threshold on the mean in-degree becomes less pronounced as α becomes larger.
(j)-(l): The mean performance of each agent plotted against his/her ability in the initial random networks (the gray boxes) and in the evolved networks with different rewiring thresholds θ =0.5 (blue circles) and θ =0.6 (red crosses). The mean performance increases linearly with ability for all values of α. As α increases, the effect of the adaptive rewiring (the difference between gray and colored points) and that of rewiring thresholds (the difference between blue and red points) on the mean performance become less pronounced.
The last two rows show the mean, (m)-(o), and the standard deviation (SD), (p)-(r), of group performance in the evolved networks plotted against the rewiring threshold. The dashed lines represent those in the initial random network. Both mean and standard deviation of group performance are higher than that in the initial random network, and show monotonic decrease with the rewiring threshold for values of α. The effects of the rewiring threshold on the mean and the SD of group performance become less pronounced as α becomes larger.
